# Structural evolution and strain generation of derived-Cu catalysts during CO_2_ electroreduction

**DOI:** 10.1038/s41467-022-32601-9

**Published:** 2022-08-18

**Authors:** Qiong Lei, Liang Huang, Jun Yin, Bambar Davaasuren, Youyou Yuan, Xinglong Dong, Zhi-Peng Wu, Xiaoqian Wang, Ke Xin Yao, Xu Lu, Yu Han

**Affiliations:** 1 Advanced Membranes and Porous Materials Center, Physical Sciences and Engineering Division, King Abdullah University of Science and Technology (KAUST), Thuwal, 23955-6900 Saudi Arabia; 2 Clean Combustion Research Center, KAUST, Thuwal, 23955-6900 Saudi Arabia; 3 KAUST Solar Center, KAUST, Thuwal, 23955-6900 Saudi Arabia; 4 Department of Applied Physics, The Hong Kong Polytechnic University, Hung Hom, Kowloon, 999077 Hong Kong, PR China; 5 Imaging and Characterization Core Lab, KAUST, Thuwal, 23955-6900 Saudi Arabia; 6 KAUST Catalysis Center, KAUST, Thuwal, 23955-6900 Saudi Arabia; 7 Multi-scale Porous Materials Center, Institute of Advanced Interdisciplinary Studies, & School of Chemistry and Chemical Engineering, Chongqing University, Chongqing, 400044 PR China

**Keywords:** Electrocatalysis, Electrochemistry, Electrocatalysis

## Abstract

Copper (Cu)-based catalysts generally exhibit high C_2+_ selectivity during the electrochemical CO_2_ reduction reaction (CO_2_RR). However, the origin of this selectivity and the influence of catalyst precursors on it are not fully understood. We combine *operando* X-ray diffraction and *operando* Raman spectroscopy to monitor the structural and compositional evolution of three Cu precursors during the CO_2_RR. The results indicate that despite different kinetics, all three precursors are completely reduced to Cu(0) with similar grain sizes (~11 nm), and that oxidized Cu species are not involved in the CO_2_RR. Furthermore, Cu(OH)_2_- and Cu_2_(OH)_2_CO_3_-derived Cu exhibit considerable tensile strain (0.43%~0.55%), whereas CuO-derived Cu does not. Theoretical calculations suggest that the tensile strain in Cu lattice is conducive to promoting CO_2_RR, which is consistent with experimental observations. The high CO_2_RR performance of some derived Cu catalysts is attributed to the combined effect of the small grain size and lattice strain, both originating from the in situ electroreduction of precursors. These findings establish correlations between Cu precursors, lattice strains, and catalytic behaviors, demonstrating the unique ability of *operando* characterization in studying electrochemical processes.

## Abstract (web summary)

Copper catalysts derived from oxidized precursors typically exhibit high selectivity for CO_2_ electroreduction to multicarbon products, yet the influencing factors that control the selectivity need further investigation. Here, the authors reveal that the high selectivity stems from small grain size and lattice strain due to in situ reduction of precursors.

## Introduction

Electrocatalytic CO_2_ reduction reaction (CO_2_RR) provides a versatile means of storing energy in chemical bonds while closing the anthropogenic carbon cycle^1^. Although significant progress has been made in the generation of single-carbon (C_1_) products (e.g., carbon monoxide, formate, methane, and methanol), in which a product selectivity of above 80% and an industrial-level current density have been achieved^2–5^, the production of valuable multicarbon (C_2+_) products (e.g., ethylene, ethanol, and n-propanol) using CO_2_RR has remained a challenge^6^.

To date, Cu-based catalysts are the main force for the production of C_2+_ products, owing to the *CO adsorption energy on Cu that favors the C–C coupling. Derived Cu catalysts, formed from the in situ reactions of oxides, hydroxides, or other oxidized Cu precursors under the reducing potentials of CO_2_RR, have attracted significant attention because they typically exhibit high selectivities toward C_2+_ products^7–9^. Although the Pourbaix diagram of Cu indicates that oxidized Cu precursors should be readily reduced to Cu(0) at negative potentials^10^, some experimental and theoretical studies have stated that Cu^+^ species or mixed oxidation states of Cu (e.g., Cu^2+^, Cu^+^, and Cu) are present in oxide- or hydroxide-derived Cu electrodes and are responsible for their high C_2+_ selectivity^8,11–16^. For example, Nilsson et al. combined spectroscopy and microscopy techniques to unravel the presence of residual oxygen in oxide-derived Cu electrocatalysts under CO_2_RR conditions^17–19^. In contrast, many other studies have demonstrated the full reduction of oxidized Cu precursors to Cu(0) and attributed the enhanced C_2+_ selectivity to structural and morphological effects^20,21^, specific crystal facet exposure^7,22^, or grain boundary and low-coordinated sites^23,24^. These inconsistent conclusions indicate that identifying the active species of derived Cu catalysts and the origin of their high C_2+_ selectivity remains controversial.

Lattice strain can modulate the activity and selectivity of electrocatalysts by breaking the linear scaling relationship^25^. Using density functional theory (DFT), Mavrikakis et al. first correlated metal lattice strain, *d*-band center shift, and adsorption energy to explain catalytic behaviors^26^. Various approaches have been employed to induce strain in Cu catalysts, including the formation of bimetallic nanoparticles^27,28^, epitaxial growth of thin films^29,30^, and crystal morphology engineering^31^. However, few studies have investigated strain effects in derived Cu catalysts for CO_2_RR. Li et al. observed microstrains in oxide-derived Cu, but they did not correlate them with CO_2_RR activity or selectivity^32^. Moreover, the influence of precursor materials on the catalytic performance of derived Cu catalysts has not been investigated. A better understanding of these aspects would facilitate the rational design of catalysts to achieve higher C_2+_ product selectivity.

Owing to the high sensitivity of Cu species to O_2_ and their immediate re-oxidation when the reducing potential is lifted^33^, *operando* characterizations are essential for probing the active species and dynamic evolution of Cu-based catalysts during CO_2_RR^34^. Particularly, some *operando* techniques, such as Raman spectroscopy, can detect the intermediates and products of CO_2_RR in real time, which has significantly facilitated the investigation of the reaction mechanism and catalyst optimization^35,36^. However, using *operando* characterization techniques that are relying on high energy beamlines (e.g., X-ray diffraction (XRD) and X-ray absorption spectroscopy) has been restricted by the limited access to synchrotron radiation facilities. The feasibility of using in-house laboratory XRD for an *operando* investigation of CO_2_RR has not been fully explored.

**Fig. 1:**
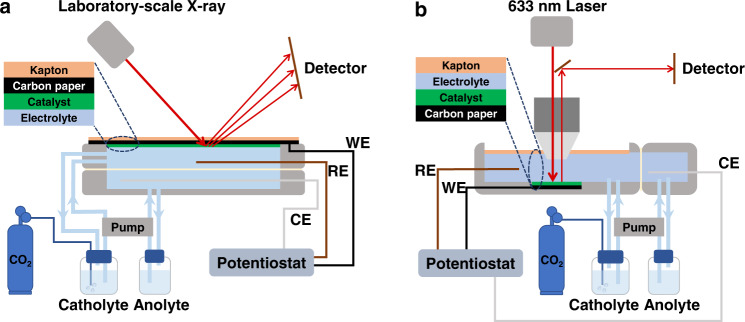
Schematic illustration of the *operando* characterization platforms. **a** X-ray diffraction (XRD) and **b** Raman spectroscopy for CO_2_ reduction reactions (CO_2_RRs) with the three investigated oxidized Cu precursors.

In this study, we investigate the structural and compositional evolution of three Cu-based catalysts derived from Cu_2_(OH)_2_CO_3_, Cu(OH)_2_ and CuO during CO_2_RR with an *operando* XRD platform that uses a laboratory-scale X-ray to analyze the phase transformation of the catalyst crystals (Fig. 1a) and *operando* Raman spectroscopy to detect the surface species (Fig. 1b). It is revealed that the three oxidized Cu precursors are all completely reduced to Cu(0) when delivering their maximum Faradaic efficiencies (FE) for C_2+_ products (${{{\mathrm{FE}}}}_{{{{\mathrm{C}}}}_{2+}}$), whereas CuO shows faster electroreduction kinetics than Cu_2_(OH)_2_CO_3_ and Cu(OH)_2_. The three derived Cu catalysts exhibit significantly higher ${{{\mathrm{FE}}}}_{{{{\mathrm{C}}}}_{2+}}$ compared to bulk Cu, which is attributed to their small grain sizes (~11 nm). *Operando* XRD discovers that Cu(OH)_2_- and Cu_2_(OH)_2_CO_3_-derived Cu exhibit obvious tensile strains, while CuO-derived Cu is almost strain-free. This finding offers a method to induce lattice strain in Cu nanocrystals by using the appropriate precursor and explains the difference in CO_2_RR activity relative to hydrogen evolution among the three derived Cu catalysts. Therefore, lattice strain has been identified as another factor promoting the CO_2_RR activity of derived Cu catalysts in addition to grain boundaries and high-index facets associated with the small grain size. These important insights cannot be gained by ex situ characterization because Cu nanocrystals undergo rapid surface oxidation and strain relaxation once electroreduction conditions are lifted. This study highlights the importance and necessity of *operando* characterizations in probing the evolution of electrocatalysts, and provides a technical solution for achieving this goal in ordinary laboratories.

## Results

### Precursor characterization

The XRD characterization revealed that the three synthesized precursors, Cu_2_(OH)_2_CO_3_, Cu(OH)_2_, and CuO, are all phase pure (Supplementary Fig. 1a), and their Raman spectra were perfectly matched with the reported standard spectra (Supplementary Fig. 2a, b)^36–39^. Scanning electron microscopy (SEM) revealed that Cu_2_(OH)_2_CO_3_ consisted of agglomerated nano-sized square rods (Supplementary Fig. 3a), whereas Cu(OH)_2_ exhibited a flower-like morphology, formed by the radial stacking of rectangular nanosheet bundles (Supplementary Fig. 3b). As the CuO sample was synthesized by heating the as-prepared Cu_2_(OH)_2_CO_3_, it retained the morphology of the parent material (Supplementary Fig. 3c) but had smaller grains, which could be attributed to the 60% atomic loss during conversion. The mean crystallite (grain) sizes of the Cu_2_(OH)_2_CO_3_, Cu(OH)_2_, and CuO nanocrystals estimated from the XRD data using the Scherrer equation were 24.7 ± 3.5, 19 ± 0.5, and 10 ± 0.5 nm, respectively (Supplementary Table 1).

### CO_2_RR performance

**Fig. 2:**
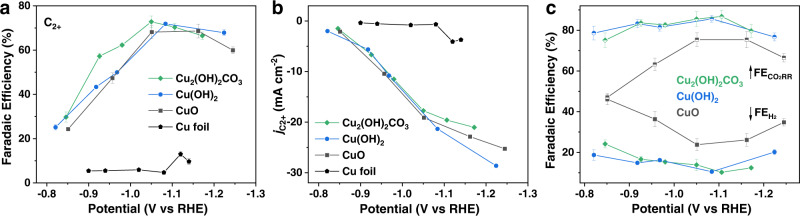
Electrocatalytic CO_2_RR performances. Comparison of **a** Faradaic efficiency of C_2+_ products and **b** C_2+_ partial current density for Cu_2_(OH)_2_CO_3_, Cu(OH)_2_, and CuO nanocrystals and Cu foil. Data are normalized using the geometric surface area. **c** Comparison of Faradaic efficiencies for overall CO_2_RR (${{{\mathrm{FE}}}}_{{{{\mathrm{CO}}}}_{2}{{{\mathrm{RR}}}}}$) and HER (${{{\mathrm{FE}}}}_{{{{\mathrm{H}}}}_{2}}$) between Cu_2_(OH)_2_CO_3_, Cu(OH)_2_, and CuO nanocrystals. All CO_2_RR experiments were performed in an H-type cell using CO_2_-saturated 0.1 M KHCO_3_ as the electrolyte for 1.5 h. Error bars represent the standard deviation of three independent measurements.

We evaluated the CO_2_RR performance of the three Cu based catalysts with an H-type cell by analyzing the gas and liquid products at different applied potentials in CO_2_-saturated 0.1 M KHCO_3_ aqueous solution in ambient conditions. The maximum ${{{\mathrm{FE}}}}_{{{{\mathrm{C}}}}_{2+}}$ of Cu_2_(OH)_2_CO_3_-, Cu(OH)_2_-, and CuO-derived Cu catalysts were 73.0, 71.9, and 68.6% at −1.05, −1.08, and −1.16 V_RHE_, respectively, substantially higher than the ${{{\mathrm{FE}}}}_{{{{\mathrm{C}}}}_{2+}}$ obtained on electropolished Cu foil (13% at −1.12 V_RHE_; Fig. 2a). The main C_2+_ products of the systems included C_2_H_4_, C_2_H_5_OH, n-C_3_H_7_OH, C_2_H_5_CHO, CH_3_CHO, and C_2_H_6_ (sequenced by decreasing FE, see Supplementary Fig. 4a–c for the detailed product distribution). The three catalysts exhibited similar C_2+_ partial current densities (${{j}}_{{{{\mathrm{C}}}}_{2+}}$) within the tested potential window, reaching −20 to −30 mA cm^−2^ at ~−1.2 V_RHE_. In contrast, the maximum ${{j}}_{{{{\mathrm{C}}}}_{2+}}$ value obtained on the Cu foil was −4.1 mA cm^−2^ at ~−1.12 V_RHE_ (Fig. 2b). The observed high C_2+_ selectivities and activities of Cu(OH)_2_- and CuO-derived Cu are consistent with findings in previous studies^7,8,40^, whereas this is the first report on the high selectivity and activity of Cu_2_(OH)_2_CO_3_-derived Cu toward C_2+_ production during CO_2_RR.

The overall FEs for CO_2_RR (${{{\mathrm{FE}}}}_{{{{\mathrm{CO}}}}_{2}{{{\mathrm{RR}}}}}$) of the three catalysts followed the order of Cu_2_(OH)_2_CO_3_ ≈ Cu(OH)_2_ > CuO (Fig. 2c). Specifically, Cu_2_(OH)_2_CO_3_- and Cu(OH)_2_-derived Cu exhibited ~75% ${{{\mathrm{FE}}}}_{{{{\mathrm{CO}}}}_{2}{{{\mathrm{RR}}}}}$ at a relatively low potential of ~−0.83 V_RHE_, whereas the ${{{\mathrm{FE}}}}_{{{{\mathrm{CO}}}}_{2}{{{\mathrm{RR}}}}}$ of CuO was only ~47% at this potential. Correspondingly, the competing hydrogen evolution reaction (HER) occurred to a significantly higher degree on CuO-derived Cu than on the other two catalysts over the tested potential range. The three catalysts exhibited a similar trend: as the potential became more negative, the FE for hydrogen (${{{\mathrm{FE}}}}_{{{{\mathrm{H}}}}_{2}}$) first decreased and then increased. The minimum ${{{\mathrm{FE}}}}_{{{{\mathrm{H}}}}_{2}}$ (i.e., the maximum ${{{\mathrm{FE}}}}_{{{{\mathrm{CO}}}}_{2}{{{\mathrm{RR}}}}}$) was reached at ~−1.1 V_RHE_, where the difference in CO_2_RR activity (or HER activity) between CuO and the other two catalysts became less significant but still discernible (Fig. 2c).

#### Redox behaviors

To further examine the catalyst evolution during CO_2_RR, we first investigated the redox behaviors of the three oxidized Cu precursors by performing Cyclic Voltammetry (CV) in 0.1 M KOH aqueous solution and Linear Sweep Voltammetry (LSV) in Ar-saturated 0.1 M of KHCO_3_ aqueous solution. The results revealed that, in both KOH and KHCO_3_ environments, the three samples undergo the same electroreduction pathway (i.e., complete reduction to Cu via the formation of Cu_2_O); however, more negative potentials are required for the electroreduction of Cu_2_(OH)_2_CO_3_ and Cu(OH)_2_ compared to CuO (see Supplementary Note and Supplementary Fig. 5 for the detailed analysis).

#### *Operando* XRD

**Fig. 3:**
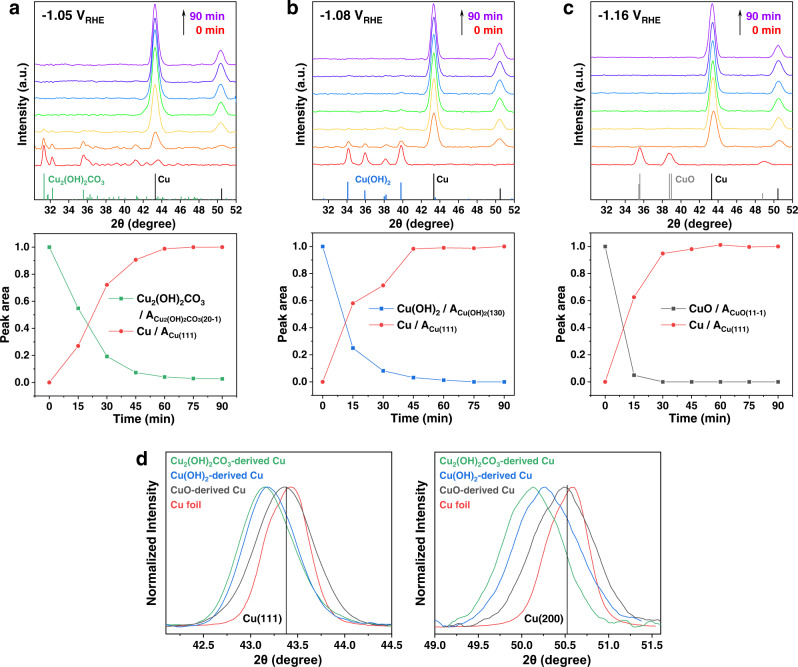
The *operando* X-ray diffraction (XRD) characterizations. Time-resolved *operando* XRD patterns (upper panel) and the corresponding quantitative peak analysis (lower panel) of **a** Cu_2_(OH)_2_CO_3_, (**b**) Cu(OH)_2_, and **c** CuO nanocrystals during the CO_2_RR at their optimum potential for C_2+_ production. Spectra were collected every 15 min. For the quantitative peak analysis, the integrated and normalized intensities of Cu_2_(OH)_2_CO_3_(20-1), Cu(OH)_2_(130), CuO(11-1) and Cu(111) were used. **d** Comparison of Cu(111) (left panel) and Cu(200) (right panel) diffraction peak position of various derived Cu and Cu foil measured from *operando* XRD experiments. Vertical lines indicate the standard diffraction peak positions.

We then performed time-resolved *operando* XRD in a customized flow cell to investigate the structural transitions of the three oxidized Cu precursors during CO_2_RR (Supplementary Figs. 6 and 7). When Cu_2_(OH)_2_CO_3_ was at its optimal C_2+_ production potential (−1.05 V_RHE_), its characteristic (20-1) peak at 31.3° gradually faded during the first 45 min of the reaction, while the fingerprint (111) and (200) peaks of metallic Cu emerged with increasing intensity after the first 15 min (Fig. 3a). No diffraction peaks related to Cu hydroxides or oxides were detected during the entire process, suggesting that these species, if present, did not form crystalline phases. Further analysis of *operando* XRD data collected at different potentials revealed the potential-dependent electroreduction kinetics of Cu_2_(OH)_2_CO_3_: the required reduction time from Cu_2_(OH)_2_CO_3_ to Cu decreased with an increase in the negativity of the applied potential (Supplementary Fig. 8a, b). Similar to Cu_2_(OH)_2_CO_3_, Cu(OH)_2_ underwent a relatively slow process before the complete reduction to metallic Cu, during which no crystalline CuO or Cu_2_O phase was detected. At its optimal potential for ${{{\mathrm{FE}}}}_{{{{\mathrm{C}}}}_{2+}}$ (−1.08 V_RHE_), the strongest (130) peak at 39.8° vanished completely after 60 min of CO_2_RR, even though metallic Cu emerged in the first 15 min (Fig. 3b). In contrast, CuO was rapidly and completely converted to metallic Cu within the first 15 min of CO_2_RR (Fig. 3c), implying faster electroreduction kinetics compared to Cu_2_(OH)_2_CO_3_ and Cu(OH)_2_. On the other hand, although the grain sizes of the starting materials were different (Supplementary Table 1), the Cu derived from the three precursors had similar grain sizes (~11 nm; estimated from the XRD data; Supplementary Table 1). The smaller mean grain sizes of the derived Cu relative to those of the parent materials could be attributed to the combined effect of the volume shrinkage of the unit-cell and crystal fragmentation^7^. We therefore concluded that the three oxidized Cu precursors, despite the differences in their chemical components and electroreduction kinetics, were completely reduced to metallic Cu with similarly small grain sizes while (or before) delivering enhanced C_2+_ selectivity under the CO_2_RR conditions.

Interestingly, Cu nanocrystals derived from Cu_2_(OH)_2_CO_3_ exhibited shifted XRD peaks relative to the Cu foil as a standard sample, implying the presence of lattice strain. Specifically, at −1.05 V_RHE_, the Cu(111) and Cu(200) diffraction peaks were shifted by ~0.25° and ~0.39° to the low-angle direction (Fig. 3d), corresponding to the tensile strains of 0.55% and 0.72%, respectively (Supplementary Table 2). This phenomenon was reproduced in measurements at different potentials, whereas the degree of strain slightly varied with the potential (Supplementary Table 2). Similarly, Cu nanocrystals derived from Cu(OH)_2_ possessed significant (0.42%−0.47%) tensile strains under CO_2_RR conditions, according to the shift in the Cu(111) diffraction peak (Fig. 3d and Supplementary Table 2). In contrast, Cu derived from CuO displayed negligible peak shifts relative to the standard regardless of the applied potential, although it had a grain size similar to the other two derived Cu samples (Fig. 3d and Supplementary Table 2). The *operando* XRD data were all corrected based on the results of a standard sample to eliminate liquid-induced peak shift effects (Supplementary Fig. 9).

#### *Operando* Raman spectroscopy

**Fig. 4:**
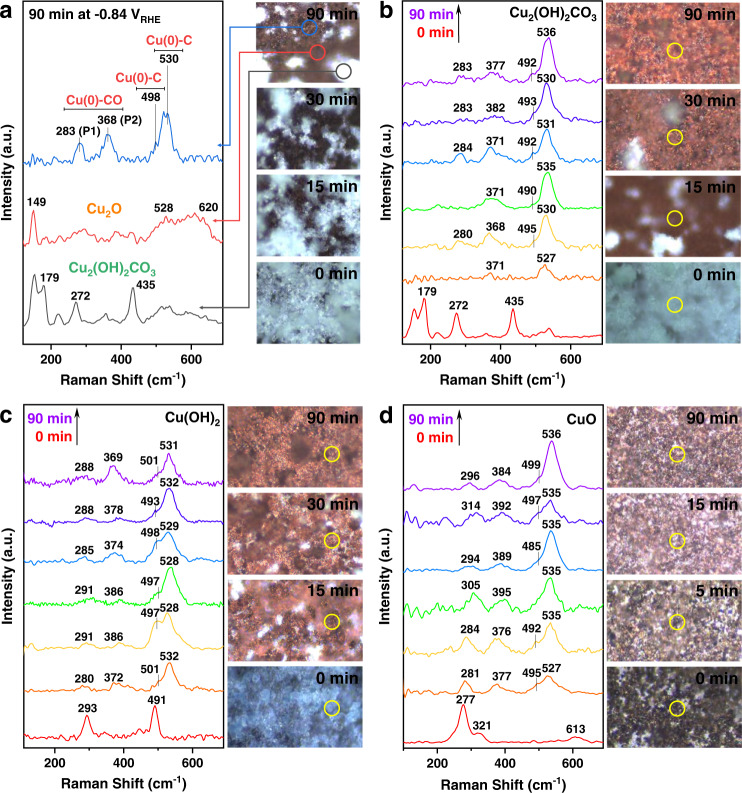
The *operando* Raman spectroscopy characterizations. **a** Time-resolved light microscopy (right panel) images of the Cu_2_(OH)_2_CO_3_ electrode surface during the CO_2_RR process at −0.84 V_RHE_, and the *operando* Raman spectra (left panel) acquired from three different areas (circled in different colors) on the Cu_2_(OH)_2_CO_3_ electrode surface after 90 min of the reaction. Time-resolved *operando* Raman spectra (left panel) and light microscopy (right panel) images of the **b** Cu_2_(OH)_2_CO_3_, (**c**) Cu(OH)_2_, and **d** CuO nanocrystals at their optimum potential for C_2+_ production during CO_2_RR. The circles indicate the locations of the laser spot with a theoretical diameter of 858 nm. Spectra were collected every 15 min.

Time-resolved *operando* Raman spectroscopy was performed in a customized flow cell to explore the oxidation state evolution of the catalyst surface during CO_2_RR (Supplementary Figs. 10 and 11). When the measurement of Cu_2_(OH)_2_CO_3_ was conducted at −0.84 V_RHE_, some areas of the electrode surface gradually changed from the initial turquoise color (the color of Cu_2_(OH)_2_CO_3_ nanocrystals) to brown after 90 min of CO_2_RR (Fig. 4a, right panel). Three types of Raman spectra were observed in different regions of the electrode surface, including the characteristic spectrum of Cu_2_(OH)_2_CO_3_ from the remaining shiny crystals and two distinct spectra from the newly formed brown area (Fig. 4a, left panel). Further analysis revealed that the red-circled region corresponded to Cu_2_O, as evidenced by the fingerprint Raman bands at 149, 528, and 620 cm^−1^^36,41^. The spectrum acquired from the blue-circled region revealed three intense bands at 283, 368, and 530 cm^−1^ and a shoulder band at 498 cm^−1^. According to recent studies, the bands at 283 and 368 cm^−1^ correspond to the frustrated rotational mode of *CO (P1) and the Cu(0)–CO stretching vibration (P2), respectively^35,41^, and the bands at 498 and 530 cm^−1^ originate from C-containing adsorbates^42–44^. Interestingly, once the negative potential of CO_2_RR was lifted, these four bands disappeared, whereas the fingerprint pattern of Cu_2_O emerged (Supplementary Fig. 12a), confirming their association with the binding intermediates to the Cu surface. These results taken together indicate that the catalyst in the blue-circled region was fully reduced to Cu(0), and the Raman bands at 283, 368, 498 (shoulder), and 530 cm^−1^ can be regarded as indicators for the formation of Cu(0) and its participation in the CO_2_RR process.

Given that the shiny crystals were confirmed to be unreduced Cu_2_(OH)_2_CO_3_, subsequent analyses were performed on the brown region of the electrode. At a more negative potential (e.g., −1.05 V_RHE_ in Fig. 4b, and −1.2 V_RHE_ in Supplementary Fig. 13), the color change of the electrode surface was faster, and few shiny crystals remained on the electrode after 30 min. Moreover, most acquired Raman spectra exhibited a similar pattern (283, 368, 498 (shoulder), and 530 cm^−1^), suggesting the predominance of Cu(0) during CO_2_RR at such negative potentials. Slight deviations within 280 to 284 and 368 to 388 cm^−1^ for the Cu(0)–CO signals can be attributed to the dynamic change in the Cu surface during CO_2_RR^45^. The other two characteristic Raman bands also shifted within 486 to 500 and 526 to 536 cm^−1^, presumably due to the variation in the C-containing species binding to the Cu surface.

*Operando* Raman measurements indicated that, similar to Cu_2_(OH)_2_CO_3_, Cu(OH)_2_ also underwent gradual and uneven electroreduction under CO_2_RR conditions (Fig. 4c). In contrast, CuO exhibited a much faster electroreduction rate. The original black electrode surface turned completely brown within the first 5 min. The Raman bands of CuO vanished within 15 min, while Cu(0) dominated the electrode surface (Fig. 4d). These observations are consistent with the bulk behaviors of the three precursors revealed through *operando* XRD.

In summary, the *operando* Raman spectroscopy echoes and complements the *operando* XRD analysis. Both methods demonstrate that the three oxidized Cu precursors (bulk and surface) are fully reduced to metallic Cu when delivering their maximum ${{{\mathrm{FE}}}}_{{{{\mathrm{C}}}}_{2+}}$ and that CuO exhibits faster reduction kinetics than the other two. Another interesting finding is that Raman signals associated with the CO_2_RR intermediates and signals of Cu_2_(OH)_2_CO_3_, Cu(OH)_2_, CuO, or Cu_2_O do not appear in the same region simultaneously. Given that XRD can only detect crystalline phases and Raman effect is weak and localized, the present data cannot completely rule out the possible presence of metastable, short-lived, amorphous oxidized Cu species in the derived Cu catalysts^16,46^.

## Discussion

### Importance and necessity of *Operando* characterizations

*Operando* characterizations provide otherwise unavailable information on the inherent states of catalysts and intermediates under reaction conditions and are particularly important for systems involving susceptible species, such as Cu. In this study, the Raman bands, which are relevant to the binding of CO_2_RR intermediates to Cu(0) (i.e., 283, 368, 498 (shoulder), and 530 cm^−1^), could not be observed through ex situ measurements due to the inevitable rapid surface oxidation of Cu (Supplementary Fig. 12a). In other words, without *operando* Raman spectroscopy, we would not have been able to determine the lack of co-occurrence of CO_2_RR intermediates and oxidized Cu species, which is crucial for determining the catalytic active species (*ubi infra*).

Likewise, significant tensile strains in Cu_2_(OH)_2_CO_3_ and Cu(OH)_2_-derived Cu could only be probed using *operando* XRD. When the electrodes containing derived Cu nanocrystals were removed from the electrochemical systems and characterized using conventional (ex situ) XRD, the Cu(111) and Cu(200) diffraction peaks appeared at their standard positions without a shift (Supplementary Fig. 12b). Moreover, a broad peak emerged at ~36.62°, which could be assigned to Cu_2_O formed through surface oxidation. We surmise that the surface oxidation of Cu nanocrystals leads to the relaxation of the lattice strain. These results demonstrate the unique ability of *operando* characterization to capture the true states of catalysts under reaction conditions.

In addition, the time-resolved *operando* characterizations revealed that the electroreduction kinetics of CuO are faster than those of Cu_2_(OH)_2_CO_3_ and Cu(OH)_2_. We note that the electroreduction behaviors of Cu_2_(OH)_2_CO_3_ and Cu(OH)_2_ under the CO_2_RR conditions have not been previously investigated using *operando* techniques, while the conclusions of several earlier studies on Cu oxides^41,47^ are consistent with the observations of the CuO sample in this work.

### Determination of active species in derived Cu catalysts

The *operando* XRD and *operando* Raman spectroscopy results collectively suggest that, despite the difference in chemical components and electroreduction kinetics, the three oxidized Cu precursors were reduced to metallic Cu while (or before) delivering enhanced C_2+_ selectivity under CO_2_RR conditions. For Cu_2_(OH)_2_CO_3_ and Cu(OH)_2_ with slow electroreduction kinetics, the temporary presence of mixed Cu oxidation states (Cu^2+^ from Cu_2_(OH)_2_CO_3_ or Cu(OH)_2_, Cu^+^ from Cu_2_O, and metallic Cu) was observed; however, Raman signals related to CO_2_RR intermediates (283, 368, 498 (shoulder), and 530 cm^−1^) were only observed in regions without Raman fingerprints of Cu_2_(OH)_2_CO_3_, Cu(OH)_2_, CuO, or Cu_2_O. These results rule out the participation of these oxidized Cu species in CO_2_RR; thus, we conclude that Cu(0) is the catalytic active site responsible for enhanced C_2+_ selectivity.

Based on this understanding, we speculate that, at the initial stage of the reaction, CuO is more selective for C_2+_ products than Cu_2_(OH)_2_CO_3_ because CuO can be reduced to Cu(0) in a shorter time. To verify this speculation, we re-examined the Cu_2_(OH)_2_CO_3_ and CuO systems by monitoring the C_2+_ production rate during the CO_2_RR at optimal potentials. Because C_2_H_4_ was the main component among the C_2+_ products in these systems, we used ${{{\mathrm{FE}}}}_{{{{\mathrm{C}}}}_{2}{{{\mathrm{H}}}}_{4}}$ as the indicator for C_2+_ selectivity. The results revealed that the ${{{\mathrm{FE}}}}_{{{{\mathrm{C}}}}_{2}{{{\mathrm{H}}}}_{4}}$ plateau was achieved faster in the CuO system than in the Cu_2_(OH)_2_CO_3_ system, although the final ${{{\mathrm{FE}}}}_{{{{\mathrm{C}}}}_{2}{{{\mathrm{H}}}}_{4}}$ values of the two systems were similar. At sampling points of 10, 15, and 20 min, CuO exhibited a higher ${{{\mathrm{FE}}}}_{{{{\mathrm{C}}}}_{2}{{{\mathrm{H}}}}_{4}}$ than Cu_2_(OH)_2_CO_3_ (Supplementary Fig. 14). This result agrees with the expectation, confirming that Cu(0) plays a key role in enhancing C_2+_ selectivity.

If Cu(0) is indeed the origin of high C_2+_ selectivity and oxidized Cu precursors are reduced to metallic Cu during CO_2_RR, the marked variation in the performance of various Cu catalysts reported in the literature need to be understood. For instance, why do hydroxide- or oxide-derived Cu catalysts generally exhibit higher C_2+_ selectivity than bulk Cu catalysts (e.g., Cu foil)? In a previous study, we demonstrated that an oxidation–reduction cycle resulted in the fragmentation of original Cu material into smaller irregular grains^7^, leading to the exposure of a variety of grain boundaries and high-index facets promoting C–C couplings for high ${{{\mathrm{FE}}}}_{{{{\mathrm{C}}}}_{2+}}$^32,48–53^. We therefore postulate that the C_2+_ selectivity is roughly related to the mean grain (crystallite) size of the Cu catalyst, and is benefited from grains that are more irregular with a decreased size.

**Fig. 5:**
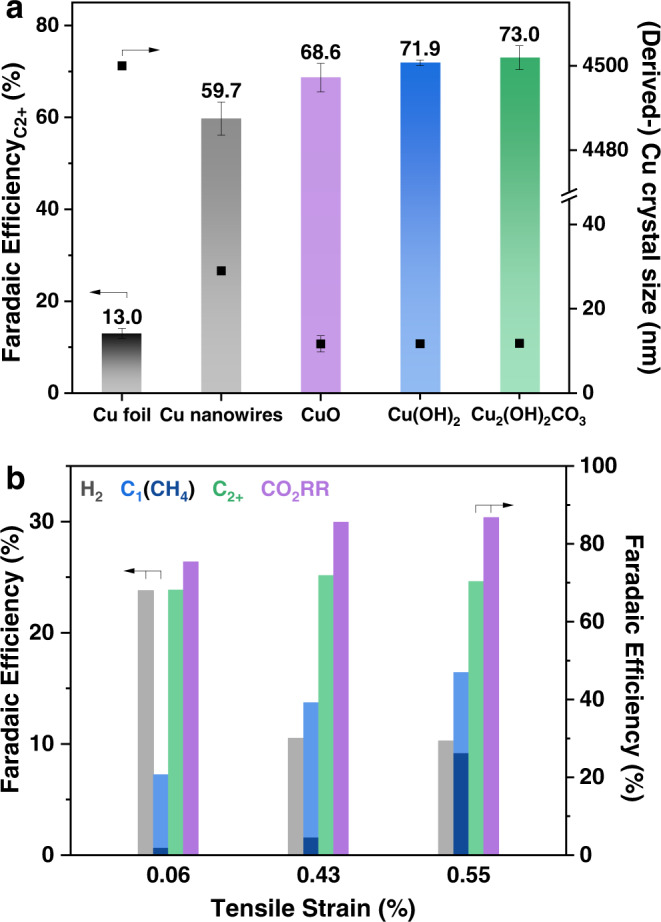
Size and strain effects on CO_2_RR behaviors of Cu-based catalysts. **a** Maximum Faradaic efficiencies for C_2+_ products and the crystal sizes of the derived Cu (from Cu_2_(OH)_2_CO_3_, Cu(OH)_2_, and CuO nanocrystals), Cu nanowires, and polycrystalline Cu foil. **b** Different tensile strain values of the derived Cu (along the [111] direction) and the corresponding Faradaic efficiencies for H_2_, C_1_, CH_4_, C_2+_ products, and the overall CO_2_RR at ~−1.1 V_RHE_. Samples with 0.06%, 0.43%, and 0.55% tensile strains represent the Cu derived from CuO, Cu(OH)_2_, and Cu_2_(OH)_2_CO_3_, respectively. Error bars represent the standard deviation of three independent measurements.

In this study, the three derived Cu catalysts with similar grain sizes (~11 nm) indeed exhibited similar ${{{\mathrm{FE}}}}_{{{{\mathrm{C}}}}_{2+}}$ (68%–73%). To further verify the correlation between the Cu grain size and C_2+_ selectivity, two additional Cu samples were prepared: Cu nanowires and electropolished polycrystalline Cu foil (Supplementary Figs. 1b and 3d–f). The mean grain size of the Cu nanowires estimated by XRD was ~29 nm, and the transmission electron microscopy revealed that the Cu foil comprised micron-sized grains (Supplementary Fig. 3e). The CO_2_RR performance of Cu nanowires and electropolished polycrystalline Cu foil was also evaluated (Supplementary Fig. 4d–e). Comparisons of the maximum ${{{\mathrm{FE}}}}_{{{{\mathrm{C}}}}_{2+}}$ and the mean grain sizes of the five tested catalysts confirmed a negative correlation between these two factors; that is, the C_2+_ selectivity increased with a decreasing Cu grain size (Fig. 5a). Another consequence of reducing the Cu grain size is an increase in the electrochemically active surface area of the electrode (Supplementary Table 1), which typically results in enhanced apparent activity in terms of current density per unit electrode area^54^.

### Strain effects

Although the three derived Cu catalysts exhibited similar C_2+_ selectivity, a discernible difference exists in the overall Faradaic efficiency for CO_2_RR (${{{\mathrm{FE}}}}_{{{{\mathrm{CO}}}}_{2}{{{\mathrm{RR}}}}}$) between CuO and the other two catalysts (CuO < Cu_2_(OH)_2_CO_3_ ≈ Cu(OH)_2_; Fig. 2c). Moreover, CuO-derived Cu is strain-free, whereas the other two derived Cu samples show significant tensile strains under CO_2_RR conditions (Fig. 3d). This coincidence led to wondering whether lattice strain is another factor affecting the catalytic performance of Cu in addition to grain boundaries and high-index facets associated with the small grain size.

**Fig. 6:**
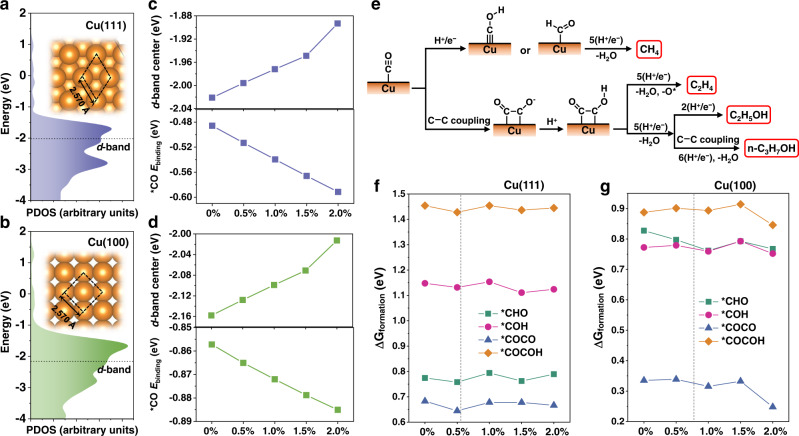
The DFT calculation results. Projected density of states (PDOS) of the *d*-band for **a** Cu(111) and **b** Cu(100) without strain. The horizontal black dashed lines indicate positions of the *d*-band center (referenced to the Fermi level). The Fermi level was set as zero. Insets are top views of the corresponding crystal structure slabs. Calculated *d*-band centers and binding energies of *CO for **c** Cu(111) and **d** Cu(100) with different strain values (0.0%, 0.5%, 1.0%, 1.5% and 2.0%). For Cu(111), the *CO coverage is 0.25 monolayer (ML) with a cross-sectional area of 9.15 × 10^−15^ cm^2^; for Cu(100), the *CO coverage is 0.11 ML with a cross-sectional area of 1.20 × 10^−14^ cm^2^. **e** Reaction pathway for the formation of CH_4_ and typical C_2+_ products during CO_2_RR. The calculated Gibbs free energies of formation (Δ*G*_formation_) of *CHO, *COH, *COCO, and *COCOH on **f** Cu(111) and **g** Cu(100) with different strain values. The vertical grey dashed lines indicate the experimental tensile strain of 0.55% for Cu(111) and 0.72% for Cu(100). All the DFT calculations were performed at the GGA/PBE level of theory.

According to the *d*-band theory, lattice strain can trigger a shift of the *d*-band center of the metal and tailor its catalytic activity^25,26,29,30^. We performed DFT calculations to investigate how tensile strain affects the *d*-band center of Cu and the consequent binding energy (*E*_binding_) of *CO on its surface, using Cu(111) and Cu(100) with different strain values as model structures (see the computational details in the Methods). The calculations revealed that for both structures, the *d*-band center shifts up toward the Fermi Level with increasing tensile strain, thus leading to a continuous increase in the *E*_binding_ of *CO. This conclusion holds for both low (Fig. 6a–d) and high (Supplementary Fig. 15a–c) *CO surface coverages. These results imply that strained Cu has a greater promoting effect on *CO adsorption than unstrained Cu, which has been experimentally verified using a recently reported method based on Raman band analysis (Supplementary Fig. 16a–d)^55^.

According to the widely accepted CO_2_RR mechanism^56–59^, *CHO/*COH and *COCO/*COCOH are the primary intermediates for the formation of CH_4_ and C_2+_ products, respectively (Fig. 6e), while the Gibbs free energies of formation (Δ*G*_formation_) of these intermediates are indicators of the ease of the corresponding pathways. The introduction of 0.5% tensile strain slightly reduces the Δ*G*_formation_ of *CHO, *COH, *COCO, and *COCOH on Cu(111) by 0.02, 0.02, 0.04, and 0.03 eV, respectively; further increases in strain do not monotonically decrease their Δ*G*_formation_ on Cu(111) but lead to fluctuations (Fig. 6f and Supplementary Fig. 17). Introducing 0.5−1.0% tensile strain leads to a decrease in Δ*G*_formation_ for *CHO, *COH, and *COCO on Cu(100), and further increasing the strain causes their Δ*G*_formation_ to first increase and then decrease. For *COCOH on Cu(100), the Δ*G*_formation_ slightly increases when the tensile strain increases from 0.0% to 1.5%, followed by a sudden drop at 2.0% strain (Fig. 6g and Supplementary Fig. 17).

Moreover, we explored the strain effect on the HER by calculating the adsorption free energy of *H (ΔG_*H_) and water dissociation activation barriers. Regardless of strain degree and surface type, the ΔG_*H_ is uphill whereas the H_2_ removal is downhill, implying that the HER on Cu is limited by the *H formation (Supplementary Fig. 18a, b). For Cu(111), although the ΔG_*H_ decreases with increasing tensile strain, it remains considerably positive at large strain values (e.g., 0.07 eV at 2.0% strain). For Cu(100), tensile strain does not affect the ΔG_*H_. The influence of tensile strain on the water dissociation activation barrier is marginal for both surfaces (Supplementary Fig. 18c, d).

In summary, the calculations indicate that the introduction of tensile strain in Cu significantly promotes the adsorption of *CO and also slightly facilitates the subsequent hydrogenation and C–C coupling, while hardly affecting the HER. Therefore, Cu with tensile strain is expected to exhibit higher overall CO_2_RR selectivity and suppressed HER, compared to unstrained Cu. Figure 5b displays the product distributions on the three catalysts at ~−1.1 V_RHE_, in which the differences in the production of H_2_, CH_4_, and C_1_ compounds between strained (Cu(OH)_2_- and Cu_2_(OH)_2_CO_3_-derived) and unstrained (CuO-derived) Cu are clearly illustrated. In comparison with the unstrained Cu, the strained Cu exhibited higher ${{{\mathrm{FE}}}}_{{{{\mathrm{CO}}}}_{2}{{{\mathrm{RR}}}}}$, primarily contributed by the enhanced production of C_1_ compounds (e.g., CH_4_), and lower ${{{\mathrm{FE}}}}_{{{{\mathrm{H}}}}_{2}}$. This result is consistent with expectations from DFT calculations, corroborating the important role that lattice strain in Cu may play in CO_2_RR.

It is not fully understood why Cu nanocrystals derived from Cu(OH)_2_ or Cu_2_(OH)_2_CO_3_ have greater tensile strain than Cu nanocrystals derived from CuO. One possible reason is that the former nanocrystals undergo more drastic shrinkage in unit-cell volume (74% for Cu_2_(OH)_2_CO_3_, 71% for Cu(OH)_2_, and 42% for CuO; Supplementary Table 3) and more pronounced elimination of components than the latter nanocrystals during the phase transition. With this hypothesis, the degree of lattice strain in Cu crystals can be tuned by the precursors, offering an effective strategy for strain engineering.

Although the promoting effect of tensile strain on C-C coupling and suppression on the competing HER have been demonstrated theoretically^60,61^, a controversy exists in the literature on how the tensile strain in Cu affects the C_1_ products during the CO_2_RR. For instance, a recent study reported that tensile strain in Cu is unfavorable for the formation of C_1_ products^29^, which is inconsistent with our observations and the conclusions of other studies^31,61^. Another study concluded that tensile strain has different effects on the production of CH_4_, depending on the thickness of the Cu layer^30^. These discrepancies imply that the lattice strain is not the primary factor determining CO_2_RR selectivity, and its effect may be overwhelmed by other coexisting factors, such as crystal size, grain boundary, and alloying effect. In this study, the high C_2+_ selectivity of derived Cu catalysts is primarily attributed to the grain boundaries and high-index facets due to the size effect, while the overall CO_2_RR selectivity (influenced by the competition between C_1_ production and HER) can be further improved by introducing tensile strain through the choice of precursors.

In summary, the as-prepared Cu_2_(OH)_2_CO_3_, Cu(OH)_2_, and CuO nanocrystals delivered an ${{{\mathrm{FE}}}}_{{{{\mathrm{C}}}}_{2+}}$ of 73.0%, 71.9%, and 68.6%, respectively, at approximately −1.1 V_RHE_ during the CO_2_RR. The time-resolved *operando* XRD, which uses a laboratory-scale X-ray source for characterizing CO_2_RR electrocatalysts, and *operando* Raman spectroscopy confirmed the full reduction of the precursors to Cu(0) while (or before) delivering enhanced C_2+_ selectivity under CO_2_RR conditions, and revealed that the electroreduction kinetics of Cu_2_(OH)_2_CO_3_ and Cu(OH)_2_ were significantly slower than those of CuO. The results collectively demonstrated that the active species responsible for the enhanced C_2+_ selectivity of the derived Cu electrodes was Cu(0), rather than oxidized Cu species. This conclusion was further verified by the faster reduction of CuO to Cu and the corresponding higher ${{{\mathrm{FE}}}}_{{{{\mathrm{C}}}}_{2}{{{\mathrm{H}}}}_{4}}$ at the initial stage of CO_2_RR compared to that of Cu_2_(OH)_2_CO_3_. However, it is worth noting that the possible presence of dynamic amorphous oxidized Cu species in the catalyst cannot be completely excluded due to the inherent limitations of XRD and Raman characterization.

A negative correlation between the Cu grain size and C_2+_ selectivity was established and the small grain size of the derived Cu (~11 nm), which resulted in an increased exposure of grain boundaries and high-index facets to facilitate the C–C coupling reaction, was considered as the primary factor for achieving high C_2+_ selectivity. *Operando* XRD revealed remarkable tensile strains in Cu nanocrystals derived from Cu(OH)_2_ and Cu_2_(OH)_2_CO_3_ but not in Cu nanocrystals derived from CuO. Theoretical calculations indicate that tensile strain in the Cu lattice helps promote the hydrogenation of *CO and C–C coupling, enhancing the overall CO_2_RR selectivity and suppressing HER, which explains the diverse performance of the three derived Cu catalysts. Reducing crystallite size and inducing lattice strain were therefore identified as two effective approaches to enhancing the performance of Cu catalysts for CO_2_RR.

The findings of this study highlight the significant role of *operando* characterization techniques in probing catalyst evolution during CO_2_RR, and the customized *operando* platforms can be readily adapted to other important electrochemical reactions, including but not limited to hydrogen/oxygen evolution, nitrogen reduction, and methane oxidation.

## Methods

### Precursor preparation

#### *Cu*_*2*_*(OH)*_*2*_*CO*_*3*_*nanocrystals and CuO nanocrystals*

First, aqueous solutions of 10 mL of 0.5 M CuSO_4_ (Sigma–Aldrich, 98%) and 12 mL of 0.5 M Na_2_CO_3_ (Sigma–Aldrich, ≥ 99.0%) were prepared at room temperature using Milli-Q water (18.2 MΩ·cm^−1^). Subsequently, the Na^+^ solution was added to the Cu^2+^ solution, and the mixture was continuously stirred. After 15 min, the mixture was heated at 60 °C and stirred for another 15 min. Afterward, the mixture was allowed to stand until fully precipitated. Then, the Cu_2_(OH)_2_CO_3_ nanocrystals were obtained via vacuum filtration. The final product was dried in a vacuum oven overnight at 30 °C. The as-prepared Cu_2_(OH)_2_CO_3_ nanocrystals were heated in a vacuum oven for 4 days at 250 °C to synthesize CuO nanocrystals.

#### *Cu(OH)*_*2*_*nanocrystals*

First, 50 mL of 0.05 M aqueous solution of Cu(NO_3_)_2_ was prepared at room temperature. Subsequently, 1 mL of 25% ammonia solution was slowly added into the solution, followed by 1 mL of 0.01 M NaOH aqueous solution under stirring. The mixture was heated to 60 °C for 15 min and then cooled to room temperature. Then, the mixture was subjected to centrifugation and copious washing with water to a neutral pH to obtain the resulting bluish Cu(OH)_2_ nanocrystals. The final product was dried in a vacuum oven overnight at 30 °C.

#### *Cu nanowires and electropolished Cu foil*

The Cu nanowires were sequentially prepared by mixing 20 mL of 15 M NaOH, 1 mL of 0.1 M Cu(NO_3_)_2_, 0.15 mL of ethylenediamine, and 0.025 mL of 35 wt% hydrazine under continuous stirring. Subsequently, the mixture was heated to 80 °C for 60 min, and the product was collected by centrifugation and washed with a 3 wt% aqueous hydrazine solution. The Cu nanowires were dried in a vacuum oven overnight at 30 °C and stored under an Ar atmosphere to minimize oxidation. Polycrystalline Cu foil was electropolished in 85% phosphoric acid (Scharlau, 85% in water) at 3.0 V vs. another Cu foil for 5 min.

### Ex situ material characterization

The chemical compositions of the precursors were investigated using powder XRD (Bruker, D8 Advance) with a Cu Kα radiation wavelength of 0.154184 nm. The obtained peaks were indexed using standard patterns (PDF#00-004-0836 for Cu, PDF#00-048-1548 for CuO, PDF#04-009-4366 for Cu(OH)_2_, and PDF#00-002-0345 for Cu_2_(OH)_2_CO_3_). The mean crystallite sizes of the precursors were estimated using the Scherrer equation ($\tau=K\lambda /\beta {\cos }\theta$, where *τ* is the mean size of the crystalline domains, *K* is the dimensionless shape factor (0.89), *λ* is the X-ray wavelength, *β* is the line broadening at half the maximum intensity (full width at half maximum), and *θ* is the Bragg angle). Ex situ Raman spectra of the samples were recorded using a WITec apyron system equipped with a 633 nm laser. Considering that the three compounds were easily reduced to Cu_2_O under the Raman laser (Supplementary Fig. 19)^36^, a low magnification objective lens (Zeiss EC Epiplan–Neofluar Dic 10X/0.25) was used and the laser power was set to 20 mW to prevent the laser-induced thermo-reduction of the samples. The surface morphologies of the samples were characterized using SEM (FEI, Magellan) at a working distance of 4 mm. All samples were coated with a layer of 3 nm of Ir prior to SEM imaging to eliminate charging caused by their poor conductivity.

### Electrode preparation

First, 20 mg of the as-prepared precursor powder was mixed with 50 µL of Nafion solution and 3 mL of ethanol. Subsequently, the mixture was sonicated for at least 30 min to obtain a homogenous ink. Next, the electrodes were prepared by airbrushing the precursor ink onto a glassy carbon substrate with an area of 1.0 × 0.5 cm^2^. For *operando* characterizations, carbon paper (Freudenberg H14C9, Fuel Cell Store) was used as the substrate. The mass loading of the precursor was approximately 0.6 mg cm^−2^.

### Electroreduction of CO_2_

The electrochemistry experiments were performed in a gas-tight H-type cell using a CHI 760e workstation. The cathode and anode compartments were separated using a Nafion 117 membrane (Fuel Cell Store). A Pt foil was used as the counter electrode, and an Ag/AgCl electrode filled with saturated KCl solution (CHI, 111) was used as the reference electrode. All potentials measured against Ag/AgCl were converted to the reversible hydrogen electrode (RHE) scale using *E*_RHE_ = *E*_Ag/AgCl_ + 0.197 V + 0.0591 × pH. The cell resistance was determined using a current-interrupt method, and the potential was manually corrected after each measurement. In addition, a 0.1 M KHCO_3_ (Honeywell, 99.5% to 101.0% (acidimetric)) aqueous solution was used as the electrolyte and was saturated with CO_2_ prior to each CO_2_RR experiment. The pH value of the cell measured using an Orion 5 star benchtop multiparameter (Thermo Scientific) was 6.8. During the CO_2_RR experiment, 5.0 sccm of CO_2_ was continuously purged into the cell, and the gaseous products were routed into an online gas chromatography (GC) system (Kechuang GC2002).

### Product analysis

Gaseous products were detected using the online GC equipped with three detectors: one thermal conductivity detector for detecting H_2_, one flame ionization detector for detecting hydrocarbons, and another flame ionization detector coupled with a methanizer (Kechuang) for detecting CO. Quantitative determination was conducted using calibration curves from standard gases.

Liquid products were analyzed using proton nuclear magnetic resonance (NMR, Bruker, 600 MHz) with a presaturation sequence for suppressing the water peak. Calibration curves were developed using commercial chemicals with 1.67 ppm dimethyl sulfoxide (Sigma–Aldrich, 99.9%) as the internal standard. In addition, FE was calculated using ${FE}=([A]\cdot V\cdot ({nF}))/Q$, where [*A*] is the analyte concentration determined using quantitative NMR, *V* represents the NMR sample volume (typically, 600 μL), *n* denotes the molar ratio of the transferred electrons, *F* indicates the Faraday constant, and *Q* denotes the total charge passed.

### *Operando* XRD

*Operando* XRD measurements were performed using a Bruker D8 Discover XRD spectrometer equipped with an IµS microfocus X-ray source and Eiger 2D detector. A 2 mm collimator was used to enhance the X-ray intensity on a small spot on the electrode. The 2D detector was vertically positioned to enable the coverage of 21.7° (2θ) in the still scan mode to cover the desired diffraction angle range at a high resolution (Supplementary Fig. 20). In addition, an optical laser-video camera system was incorporated to enable easy and accurate sample positioning and system alignment. A customized electrochemical cell (Supplementary Fig. 6) was used with a Pt wire and an Ag/AgCl electrode (CHI 111; saturated KCl solution) as the counter and reference electrodes, respectively. The cathode and anode compartments were separated using a Fumasep FKB-PK-130 membrane (Fuel Cell Store). The precursors were airbrushed onto one side of the carbon paper and positioned close to the glassy carbon on the cell to function as the working electrode. The electrolyte (50 mL of 0.1 M KHCO_3_) was saturated with CO_2_ while circulating through the cell at 1 mL min^−1^. During the measurements, the cell was fastened on the stage using psi tilted to 30° for the better release of in situ formed bubbles from the electrode surface. For the Cu_2_(OH)_2_CO_3_ nanocrystals, the measurements were conducted at −0.84, −1.05, and −1.2 V_RHE_. For Cu(OH)_2_ and CuO nanocrystals, the potential was set to −1.08 and −1.16 V_RHE_, respectively. The 2D diffraction patterns were collected in the still scan mode at an integration time of 900 s. The raw data were integrated using DIFFRAC.EVA software, and the background was removed for improved peak identification.

### Strain determination

The tensile strain values of derived Cu samples were calculated using the equation:

1 $${{{{{\rm{Tensile}}}}}}\,{{{{{\rm{strain}}}}}}\;(\%)=\frac{d-{d}_{0}}{{d}_{0}}\times 100\%$$

where *d* is the *d*-spacing of a given reflection (e.g., Cu(111) or Cu(100)) determined from the XRD pattern and *d*_*0*_ is the corresponding *d*-spacing in the standard Cu sample.

For the *operando* XRD experiments, the theta angle was set to 20.28° for still scan mode to achieve the desired diffraction angle range (30°−51.7°, 2θ) at a high resolution. Under such conditions, the illuminated area on the electrode is an ellipse of ~4.7 mm^2^. The obtained strain value is the average result of the illuminated area.

### *Operando* Raman spectroscopy

*Operando* Raman spectra were recorded using a WITec apyron system with a water-immersion objective lens (Zeiss W Plan-Apochromat 63X/1) and a 633 nm laser beam. A low laser power of 0.8 mW was used to prevent the irradiation-induced modification on the precursor surface. The measurements were conducted in a customized electrochemical cell (Supplementary Fig. 10) with a Pt wire and an Ag/AgCl electrode (CHI 111; saturated KCl solution) as the counter and reference electrode, respectively. The cathode and anode compartments were separated using a Fumasep FKB-PK-130 membrane (Fuel Cell Store). The electrolyte (50 mL of 0.1 M KHCO_3_) was saturated with CO_2_ while circulating at 1 mL min^−1^ above the electrode surface to remove bubbles produced in situ. The Raman signals were collected in a back-scattering geometry every 15 min along the CO_2_RR process. The raw data were recorded and processed using WITec Control Five software, and the background was removed to enable improved peak identification.

### Computational methods

The DFT calculations were conducted using the projector-augmented wave method, as implemented in Vienna Ab initio Simulation Package code^62,63^. The generalized gradient approximation with the Perdew–Burke–Ernzerhof exchange-correlation functional was used. A uniform 6 × 6 × 6 *k*-mesh grid in the Brillouin zone was employed to optimize the crystal structure of bulk Cu, and the resulting lattice parameter was *a* = 3.635 Å. The Cu slab model with five atomic layers had a (2 × 2) and a (3 × 3) lateral periodicity with (111) and (100) exposed surfaces, respectively, and the slab replica was separated by ∼20 Å of vacuum. The lattice parameters were set to *a* = 3.653 Å, 3.671 Å, 3.690 Å, and 3.708 Å for Cu(111) and Cu(100) slabs to mimic 0.5%, 1.0%, 1.5%, and 2.0% tensile strain, respectively. A 2 × 2 × 1 *k*-mesh was used for all Cu(111) and Cu(100) slab structures. The plane-wave basis set cutoffs of the wave functions were set at 500 eV for the bulk and 450 eV for slab structures without and with molecular adsorption. The atomic positions of all structures were fully relaxed until the forces on each atom were less than 0.01 eV/Å.

The Gibbs free energy values were calculated using the following equations:

2 $$G={E}_{{{{{{\rm{DFT}}}}}}}+{E}_{{{{{{\rm{ZPE}}}}}}}+{\int }_{0}^{298.15}{C}_{v}{dT}-{TS}$$

3 $${E}_{ZPE}=\frac{1}{2}\sum \;h\nu$$

4 $${\int }_{0}^{298.15}{C}_{v}dT={k}_{B}\sum {\left(\frac{h\nu }{{k}_{B}T}\right)}^{2}\frac{\exp \left(\frac{h\nu }{{k}_{B}T}\right)}{{\left[\exp \left(\frac{h\nu }{{k}_{B}T}\right)-1\right]}^{2}}$$

where ${E}_{{DFT}}$ denotes the energy calculated by DFT, ${E}_{{ZPE}}$ represents the vibrational zero-point energy, ${\int }_{0}^{298.15}{C}_{v}{dT}$ indicates the heat capacity, and *S* is the entropy, respectively. In addition, ℎ is the Planck constant, *k*_*B*_ denotes the Boltzmann constant, and *v* represents the vibrational frequency. Only vibrational modes were considered for calculating the entropy due to the scarce contribution from translational and rotational modes. The Gibbs free energy of formation (Δ*G*_formation_) for *COCOH is calculated as Δ*G*_formation_(*COCOH) = G_*COCOH_ – (G_*CO+*CO_ + *G*_H2/2_). In addition, Δ*G*_formation_ for *COH is calculated as Δ*G*_formation_(*COH) = G_*COH_ – (G_*CO_ + *G*_H2/2_), Δ*G*_formation_ for *CHO is calculated as Δ*G*_formation_(*CHO) = G_*CHO_ – (G_*CO_ + *G*_H2/2_), and Δ*G*_formation_ for *COCO is calculated as Δ*G*_formation_(*COCO) = G_*COCO_ – (G_*CO_ + *G*_CO_). The thermodynamic parameters for calculating Gibbs free energies are given in Supplementary Tables S4–S13.

The hydrogen adsorption free energy G_*H_ is calculated as G_*H_ = *E* (slab + H) – *E*(slab) – *E*(H_2_)/2 + Δ*E*_ZPE_ – TΔS, where *E* (slab + H) and *E*(slab) are the total energies of the Cu(111) or Cu(100) slab with and without H adsorption; *E*(H_2_) is the total energy of a H_2_ molecule; Δ*E*_ZPE_ is the difference in the zero-point energy between gas phase of H_2_ and the adsorbed H atom; and ΔS is the difference in entropy. To determine the transition state (TS) for the water dissociation on the Cu(111) and Cu(100) surfaces, the climbing image nudged elastic band (NEB) method was employed, and the force convergence tolerance on each atom was set to be 0.05 eV/Å during the search of the minimum energy path.

## Supplementary information

Supplementary Information

## Data Availability

The data supporting the findings of this study are provided in the Supplementary Information/Source Data file. The input and output files for the DFT calculations are available in the IoChem-BD database (https://iochem-bd.bsc.es/browse/review-collection/100/215881/6ca411524a36a3f2c1194679). Additional data are available from the corresponding authors on request. Source data are provided with this paper.

## Source data

Source data

## Code availability

Vienna ab initio simulation package (VASP) for the DFT calculations is available at https://www.vasp.at/.

## References

[1] Gao DF, Arán-Ais RM, Jeon HS, Roldan Cuenya B (2019). Rational catalyst and electrolyte design for CO_2_ electroreduction towards multicarbon products. Nat. Catal..

[2] Xia C (2019). Continuous production of pure liquid fuel solutions via electrocatalytic CO_2_ reduction using solid-electrolyte devices. Nat. Energy.

[3] Zheng TT (2019). Large-scale and highly selective CO_2_ electrocatalytic reduction on nickel single-atom catalyst. Joule.

[4] Lu L (2018). Highly efficient electroreduction of CO_2_ to methanol on palladium-copper bimetallic aerogels. Angew. Chem. Int. Ed. Engl..

[5] Wang YS, Chen JX, Wang GX, Li Y, Wen ZH (2018). Perfluorinated covalent triazine framework derived hybrids for the highly selective electroconversion of carbon dioxide into methane. Angew. Chem. Int. Ed..

[6] Fan L (2020). Strategies in catalysts and electrolyzer design for electrochemical CO_2_ reduction toward C_2+_ products. Sci. Adv..

[7] Lei Q (2020). Investigating the Origin of Enhanced C_2+_ Selectivity in Oxide-/Hydroxide-Derived Copper Electrodes during CO_2_ Electroreduction. J. Am. Chem. Soc..

[8] Lee SY (2018). Mixed copper states in anodized Cu electrocatalyst for stable and selective ethylene production from CO_2_ reduction. J. Am. Chem. Soc..

[9] Liang ZQ (2018). Copper-on-nitride enhances the stable electrosynthesis of multi-carbon products from CO_2_. Nat. Commun..

[10] Van Muylder, J. *Electrochemical Materials Science* Ch. 1 (Springer US: Boston, MA, 1981).

[11] Mistry H (2016). Highly selective plasma-activated copper catalysts for carbon dioxide reduction to ethylene. Nat. Commun..

[12] Fan QK (2021). Manipulating Cu nanoparticle surface oxidation states tunes catalytic selectivity toward CH_4_ or C_2+_ products in CO_2_ electroreduction. Adv. Energy Mater..

[13] Yang PP (2020). Protecting copper oxidation state via intermediate confinement for selective CO_2_ electroreduction to C_2+_ Fuels. J. Am. Chem. Soc..

[14] Liu C (2017). Stability and effects of subsurface oxygen in oxide-derived Cu catalyst for CO_2_ reduction. J. Phys. Chem. C..

[15] Favaro M (2017). Subsurface oxide plays a critical role in CO_2_ activation by Cu(111) surfaces to form chemisorbed CO_2_, the first step in reduction of CO_2_. Proc. Natl Acad. Sci. USA.

[16] Dattila F, García-Muelas R, López N (2020). Active and selective ensembles in oxide-derived copper catalysts for CO_2_ reduction. ACS Energy Lett..

[17] Eilert A (2017). Subsurface oxygen in oxide-derived copper electrocatalysts for carbon dioxide reduction. J. Phys. Chem. Lett..

[18] Cavalca F (2017). Nature and distribution of stable subsurface oxygen in copper electrodes during electrochemical CO_2_ reduction. J. Phys. Chem. C..

[19] Wang HY (2022). Direct evidence of subsurface oxygen formation in oxide-derived Cu by X-ray photoelectron spectroscopy. Angew. Chem. Int. Ed..

[20] Scholten F, Nguyen KC, Bruce JP, Heyde M, Roldan Cuenya B (2021). Identifying structure-selectivity correlations in the electrochemical reduction of CO_2_: A comparison of well-ordered atomically clean and chemically etched copper single-crystal surfaces. Angew. Chem. Int. Ed. Engl..

[21] Kim T (2018). Enhancing C_2_–C_3_ production from CO_2_ on copper electrocatalysts via a potential-dependent mesostructure. ACS Appl. Energy Mater..

[22] Jiang K (2018). Metal ion cycling of Cu foil for selective C-C coupling in electrochemical CO_2_ reduction. Nat. Catal..

[23] Möller T (2020). Electrocatalytic CO_2_ reduction on CuO_x_ Nanocubes: Tracking the evolution of chemical state, geometric structure, and catalytic selectivity using Operando spectroscopy. Angew. Chem. Int. Ed. Engl..

[24] Velasco-Velez JJ (2020). Revealing the active phase of copper during the electroreduction of CO_2_ in aqueous electrolyte by correlating in situ X-ray spectroscopy and in situ electron microscopy. ACS Energy Lett..

[25] Jansonius RP, Reid LM, Virca CN, Berlinguette CP (2019). Strain engineering electrocatalysts for selective CO_2_ reduction. ACS Energy Lett..

[26] Mavrikakis M, Hammer B, Nørskov JK (1998). Effect of strain on the reactivity of metal surfaces. Phys. Rev. Lett..

[27] Liu FZ, Wu C, Yang SC (2017). Strain and Ligand Effects on CO_2_ Reduction Reactions over Cu-Metal Heterostructure Catalysts. J. Phys. Chem. C..

[28] Monzó J (2015). Enhanced electrocatalytic activity of Au@Cu core@shell nanoparticles towards CO_2_ reduction. J. Mater. Chem. A.

[29] Kim T, Kumar RE, Brock JA, Fullerton EE, Fenning DP (2021). How Strain Alters CO_2_ Electroreduction on Model Cu(001) Surfaces. ACS Catal..

[30] Du MS, Zhao X, Zhu G, Hsu HY, Liu F (2021). Elastic strain controlling the activity and selectivity of CO_2_ electroreduction on Cu overlayers. J. Mater. Chem. A.

[31] Liu SG, Huang SP (2019). Structure engineering of Cu-based nanoparticles for electrochemical reduction of CO_2_. J. Catal..

[32] Li CW, Ciston J, Kanan MW (2014). Electroreduction of carbon monoxide to liquid fuel on oxide-derived nanocrystalline copper. Nature.

[33] Chen CJ (2021). The in situ study of surface species and structures of oxide-derived copper catalysts for electrochemical CO_2_ reduction dagger. Chem. Sci..

[34] Li X, Wang S, Li L, Sun Y, Xie Y (2020). Progress and perspective for in situ studies of CO_2_ reduction. J. Am. Chem. Soc..

[35] Jiang S, Klingan K, Pasquini C, Dau H (2019). New aspects of operando Raman spectroscopy applied to electrochemical CO_2_ reduction on Cu foams. J. Chem. Phys..

[36] Deng YL, Handoko AD, Du YH, Xi SB, Yeo BS (2016). In situ Raman spectroscopy of copper and copper oxide surfaces during electrochemical oxygen evolution reaction: Identification of Cu^III^ oxides as catalytically active species. ACS Catal..

[37] Marucci G, Beeby A, Parker AW, Nicholson CE (2018). Raman spectroscopic library of medieval pigments collected with five different wavelengths for investigation of illuminated manuscripts. Anal. Methods-UK.

[38] Frost RL, Martens WN, Rintoul L, Mahmutagic E, Kloprogge JT (2002). Raman spectroscopic study of azurite and malachite at 298 and 77 K. J. Raman Spectrosc..

[39] Wang W (2003). A simple wet-chemical synthesis and characterization of CuO nanorods. Appl. Phys. A.

[40] Cheng D (2021). The nature of active sites for carbon dioxide electroreduction over oxide-derived copper catalysts. Nat. Commun..

[41] Dutta A (2020). CO_2_ electrolysis - Complementary operando XRD, XAS and Raman spectroscopy study on the stability of Cu_x_O foam catalysts. J. Catal..

[42] Ren D, Gao J, Zakeeruddin SM, Grätzel M (2021). New insights into the interface of electrochemical flow cells for carbon dioxide reduction to ethylene. J. Phys. Chem. Lett..

[43] Moradzaman M, Mul G (2021). In situ Raman study of potential-dependent surface adsorbed carbonate, CO, OH, and C species on Cu electrodes during electrochemical reduction of CO_2_. Chemelectrochemistry.

[44] Shan W (2020). In situ surface-enhanced Raman spectroscopic evidence on the origin of selectivity in CO_2_ electrocatalytic reduction. ACS Nano.

[45] Schmitt KG, Gewirth AA (2014). In situ surface-enhanced Raman spectroscopy of the electrochemical reduction of carbon dioxide on silver with 3,5-Diamino-1,2,4-Triazole. J. Phys. Chem. C..

[46] Liu G (2021). CO_2_ reduction on pure Cu produces only H_2_ after subsurface O is depleted: Theory and experiment. Proc. Natl Acad. Sci. USA.

[47] Scott SB (2019). Absence of Oxidized Phases in Cu under CO Reduction Conditions. ACS Energy Lett..

[48] Feng X, Jiang K, Fan S, Kanan MW (2016). A direct grain-boundary-activity correlation for CO electroreduction on Cu nanoparticles. ACS Cent. Sci..

[49] Handoko AD (2016). Mechanistic Insights into the selective electroreduction of carbon dioxide to ethylene on Cu_2_O-derived copper catalysts. J. Phys. Chem. C..

[50] Jung H (2019). Electrochemical fragmentation of Cu_2_O nanoparticles enhancing selective C-C coupling from CO_2_ reduction reaction. J. Am. Chem. Soc..

[51] Velasco-Vélez JJ (2020). On the activity/selectivity and phase stability of thermally grown copper oxides during the electrocatalytic reduction of CO_2_. ACS Catal..

[52] Lum YW, Yue BB, Lobaccaro P, Bell AT, Ager JW (2017). Optimizing C-C Coupling on Oxide-Derived Copper Catalysts for Electrochemical CO_2_ Reduction. J. Phys. Chem. C..

[53] Chen ZQ (2020). Grain-Boundary-rich copper for efficient solar-driven electrochemical CO_2_ reduction to ethylene and ethanol. J. Am. Chem. Soc..

[54] Nitopi S (2019). Progress and Perspectives of Electrochemical CO_2_ Reduction on Copper in Aqueous Electrolyte. Chem. Rev..

[55] Zhan C (2021). Revealing the CO Coverage-Driven C-C Coupling Mechanism for Electrochemical CO_2_ Reduction on Cu_2_O Nanocubes via Operando Raman Spectroscopy. ACS Catal..

[56] Zheng Y (2019). Understanding the Roadmap for Electrochemical Reduction of CO_2_ to Multi-Carbon Oxygenates and Hydrocarbons on Copper-Based Catalysts. J. Am. Chem. Soc..

[57] De Luna P (2018). Catalyst electro-redeposition controls morphology and oxidation state for selective carbon dioxide reduction. Nat. Catal..

[58] Kortlever R, Shen J, Schouten KJP, Calle-Vallejo F, Koper MTM (2015). Catalysts and Reaction Pathways for the Electrochemical Reduction of Carbon Dioxide. J. Phys. Chem. Lett..

[59] Birdja YY (2019). Advances and challenges in understanding the electrocatalytic conversion of carbon dioxide to fuels. Nat. Energy.

[60] Sandberg RB, Montoya JH, Chan K, Nørskov JK (2016). CO-CO coupling on Cu facets: Coverage, strain and field effects. Surf. Sci..

[61] Chen ZZ, Zhang X, Lu G (2015). Overpotential for CO_2_ electroreduction lowered on strained penta-twinned Cu nanowires. Chem. Sci..

[62] Kresse G, Hafner J (1993). Ab-Initio Molecular-Dynamics for Open-Shell Transition-Metals. Phys. Rev. B.

[63] Kresse G, Furthmüller J (1996). Efficient iterative schemes for ab initio total-energy calculations using a plane-wave basis set. Phys. Rev. B.

