# Supplementary material for: Structural evolution and strain generation of derived-Cu catalysts during CO2 electroreduction
Source: Nat Commun. 2022 Aug 18;13:4857. doi: 10.1038/s41467-022-32601-9 (PMC9388520; doi:10.1038/s41467-022-32601-9)
Supplement: Supplementary file 1 — Supplementary Information [file 41467_2022_32601_MOESM1_ESM.pdf]

Supplementary Information

Structural evolution and strain generation of derived-Cu catalysts during CO_2 electroreduction

Qiong Lei,[1] Liang Huang,[2,3] Jun Yin,[1,7] Bambar Davaasuren,[4] Youyou Yuan,[4] Xinglong Dong,[1,5] Zhi-Peng Wu,[1] Xiaoqian Wang,[1] Ke Xin Yao,[6] Xu Lu,*[2,3] Yu Han*[1,5]*

[1] Advanced Membranes and Porous Materials Center, Physical Sciences and Engineering Division, King Abdullah University of Science and Technology (KAUST), Thuwal 23955-6900, Saudi Arabia.

[2] Clean Combustion Research Center, KAUST, Thuwal 23955-6900, Saudi Arabia.

[3] KAUST Solar Center, KAUST, Thuwal 23955-6900, Saudi Arabia.

[4] Imaging and Characterization Core Lab, KAUST, Thuwal 23955-6900, Saudi Arabia.

[5] KAUST Catalysis Center, KAUST, Thuwal 23955-6900, Saudi Arabia.

[6] Multi-scale Porous Materials Center, Institute of Advanced Interdisciplinary Studies, & School of Chemistry and Chemical Engineering, Chongqing University, Chongqing 400044, P. R. China.

[7] Department of Applied Physics, The Hong Kong Polytechnic University, Hung Hom, Kowloon 999077, Hong Kong, P. R. China.

Corresponding Authors

* yu.han@kaust.edu.sa, xu.lu@kaust.edu.sa, and jun.yin@polyu.edu.hk

This supplementary information contains:

- Supplementary Note
- Supplementary Figs. 1-20
- Supplementary Tables 1-13
- Supplementary References

Supplementary Note

Before addressing the catalyst evolution during the CO_2RR, we first investigated the redox behavior of the three oxidized Cu samples by performing Cyclic Voltammetry (CV) in 0.1 M KOH aqueous solution. Two reduction peaks were observed in the CV pattern of CuO at 0.57 and 0.32 V_{RHE}, corresponding to the reduction of CuO to Cu_2O and then to Cu, respectively (Supplementary Fig. 5a), which is consistent with the findings of previous studies.[1-2] The reduction peaks corresponding to the reduction of $Cu(OH)_2$ and $Cu_2(OH)_2CO_3$ to Cu_2O were maintained at approximately 0.55 V_{RHE}, whereas those corresponding to the reduction to Cu shifted to 0.11 V_{RHE} (Supplementary Figs. 5b-c). An additional peak was observed at 0.97 V_{RHE}, which could be attributed to the dissociation of $Cu(OH)_2$ or $Cu_2(OH)_2CO_3$ to Cu^{2+}. These results indicate that the electroreduction of $Cu_2(OH)_2CO_3$, $Cu(OH)_2$, and CuO goes through a Cu_2O formation stage, followed by a full reduction to Cu.

Linear Sweep Voltammetry (LSV) was performed on the three oxidized Cu samples in Ar-saturated 0.1 M $KHCO_3$ (Supplementary Fig. 5d). Two intense peaks were observed in the LSV curve of CuO at -0.16 and -0.58 V_{RHE}, which corresponded to the reduction of CuO to Cu_2O and then to Cu, respectively, whereas only one broad reduction peak was observed on $Cu_2(OH)_2CO_3$ at -0.96 V_{RHE}, and two broad reduction peaks on $Cu(OH)_2$ at -0.69 and -1.18V_{RHE}. These results indicate that in both KOH and $KHCO_3$ environments, the electroreduction of $Cu_2(OH)_2CO_3$ and $Cu(OH)_2$ to Cu required more negative potentials compared to that of CuO. It is important to note that the data presented here are the first LSV scans of each freshly prepared electrode; therefore, the current density and potential cannot directly correlate with those in the discussion of the CO_2RR performance of the catalysts, because the electroreduction kinetics of oxidized Cu phases can be slow on the time scale of CO_2RR[3] (details will be discussed in the subsequent paragraphs in the main text).

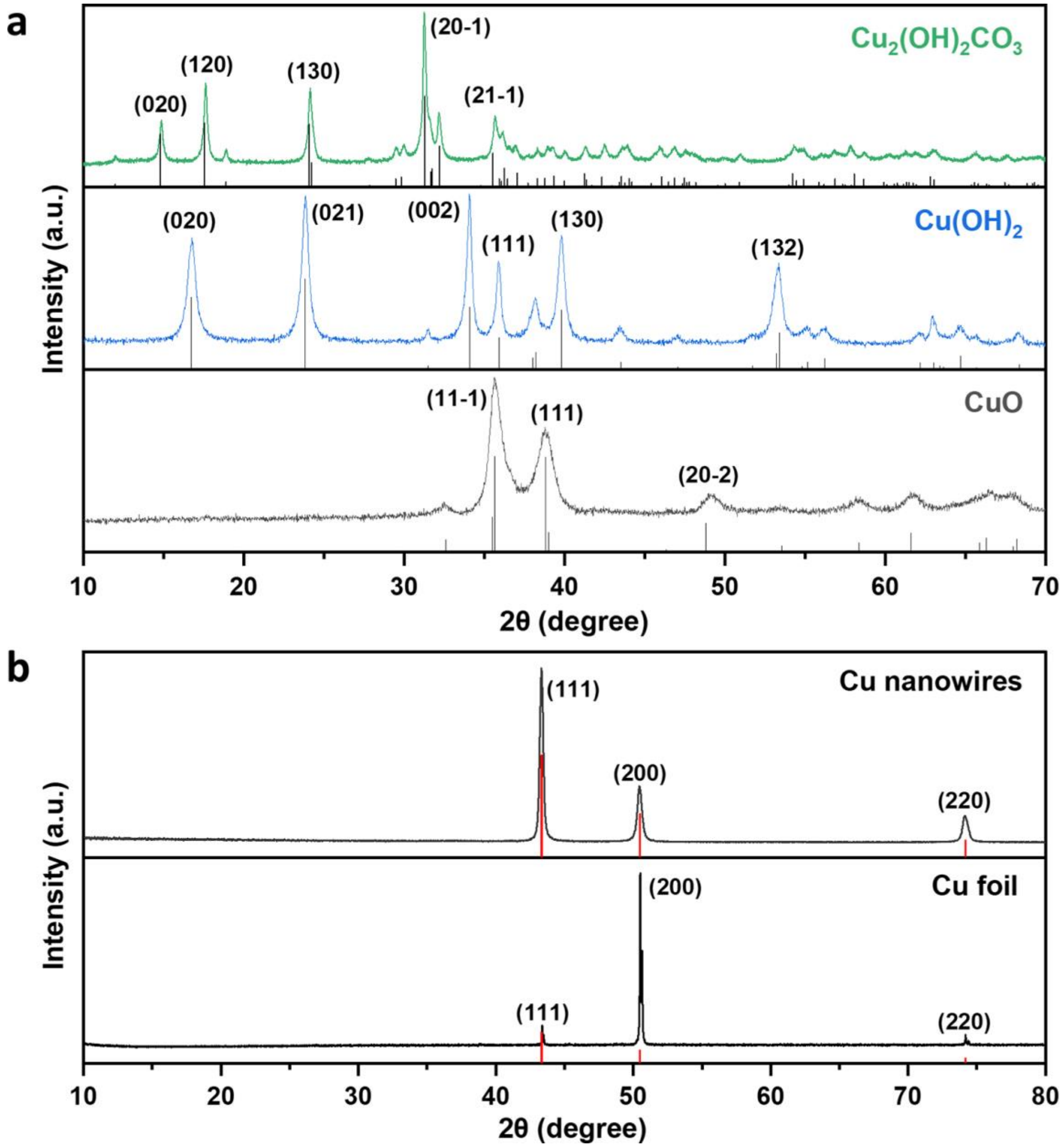

Supplementary Fig. 1. Indexed *ex situ* XRD patterns of (a) $Cu_2(OH)_2CO_3$, $Cu(OH)_2$, and CuO nanocrystals, and (b) Cu nanowires and polycrystalline Cu foil.

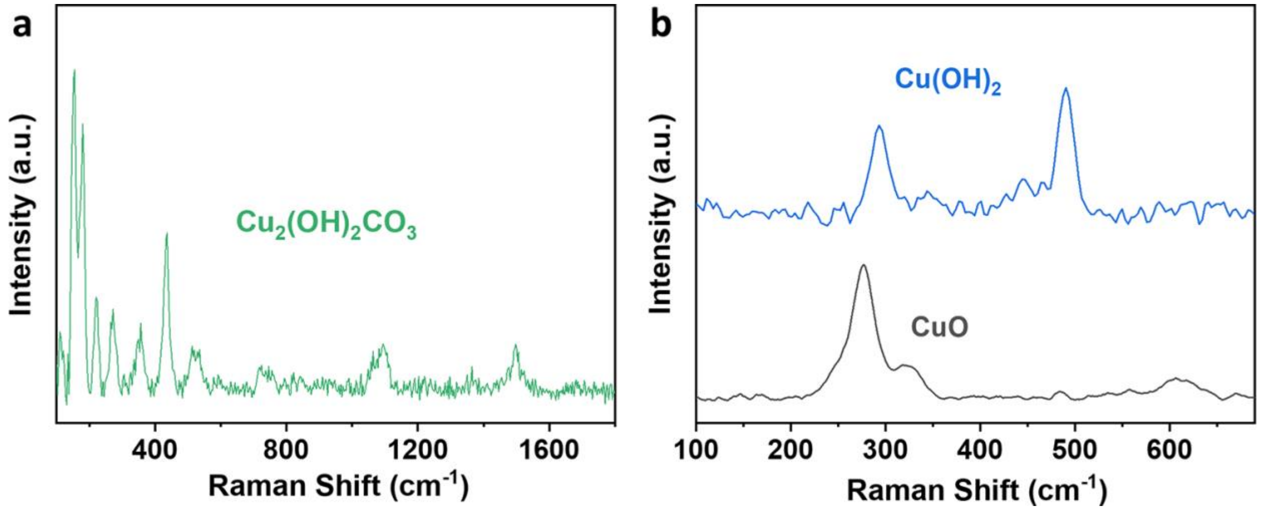

Supplementary Fig. 2. *Ex situ* Raman spectra of (a) $Cu_2(OH)_2CO_3$, and (b) $Cu(OH)_2$ and CuO nanocrystals.

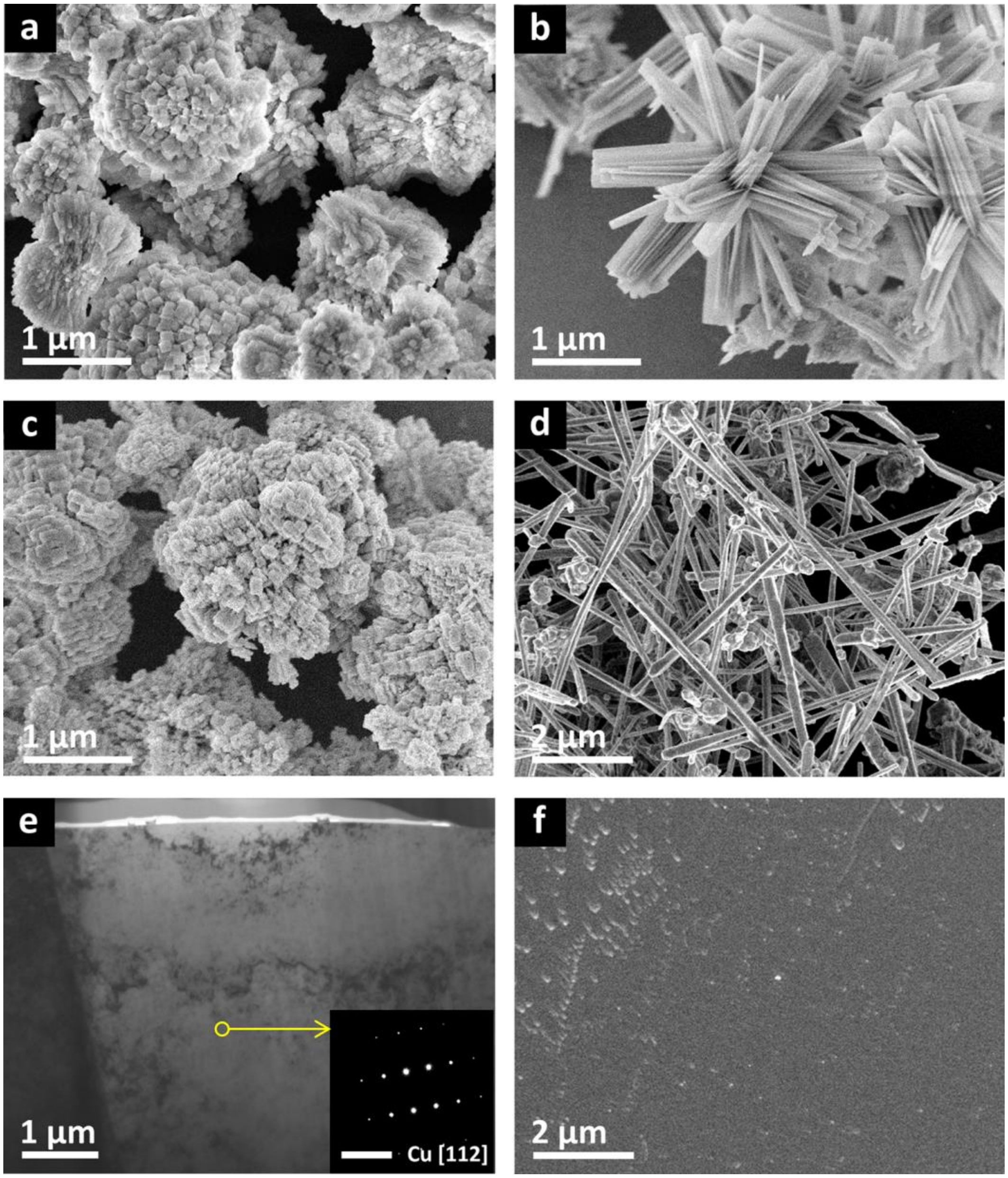

Supplementary Fig. 3. SEM images of (a) $Cu_2(OH)_2CO_3$, (b) $Cu(OH)_2$, (c) CuO nanocrystals, (d) Cu nanowires, and (f) polycrystalline Cu foil. (e) TEM image of focused ion beam (FIB)-fabricated specimen from electropolished Cu foil. The corresponding SAED pattern (inset, scale bar 10 1/nm) was collected in the circled area with a diameter of ~160 nm. FIB was performed using the standard lift-out technique on a dual-beam FIB-SEM (FEI, Helios Nanolab Dualbeam) equipped with an OmniProbe micromanipulator to prepare ultrathin (~100 nm) lamella from the electrode for TEM analysis on the cross-section. The prepared lamella was mounted to a copper half-grid and immediately transferred to glovebox for storage.

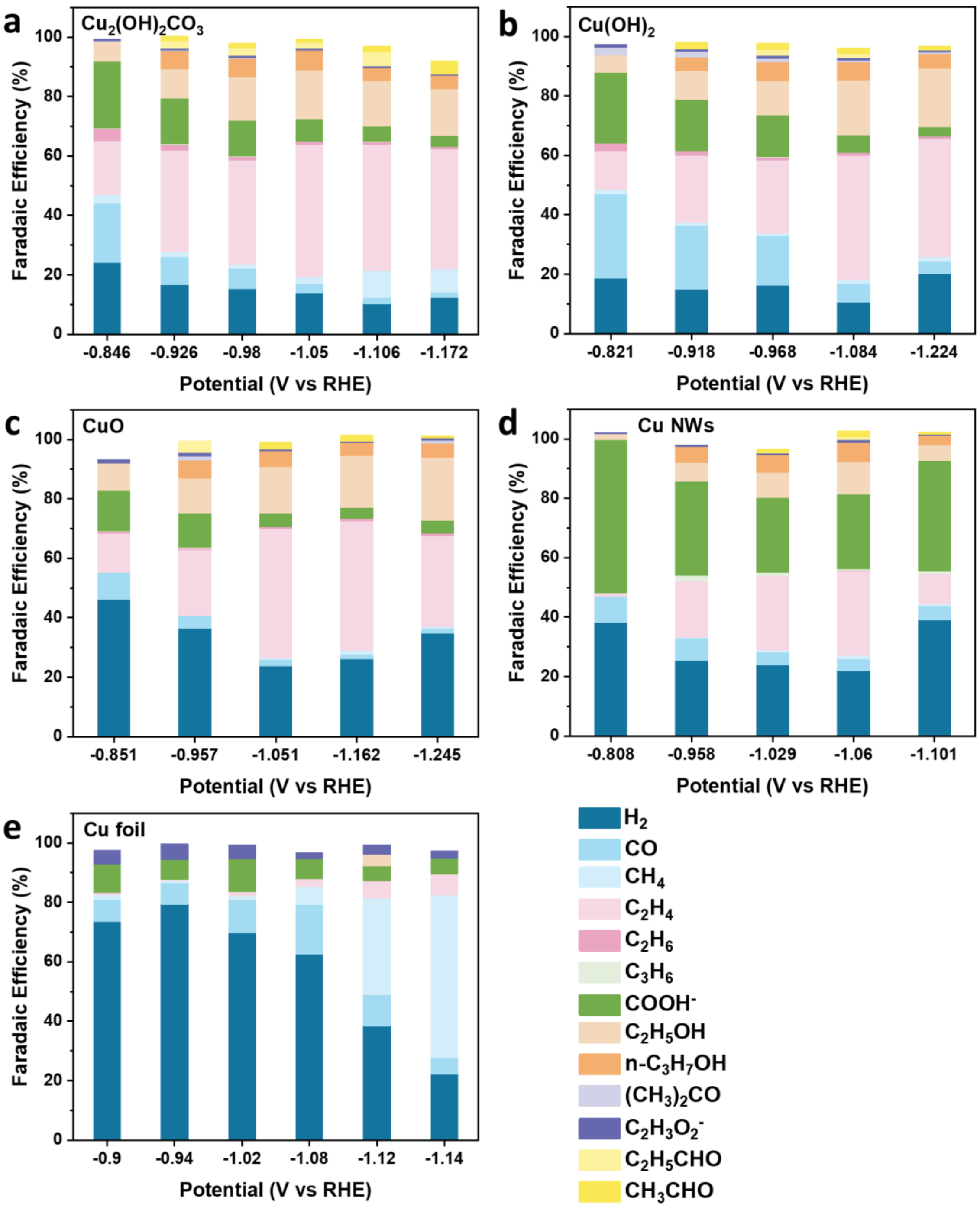

Supplementary Fig. 4. Product distribution for the CO_2RR with (a) $Cu_2(OH)_2CO_3$, (b) $Cu(OH)_2$, (c) CuO nanocrystals, (d) Cu nanowires, and (e) polycrystalline Cu foil at different potentials.

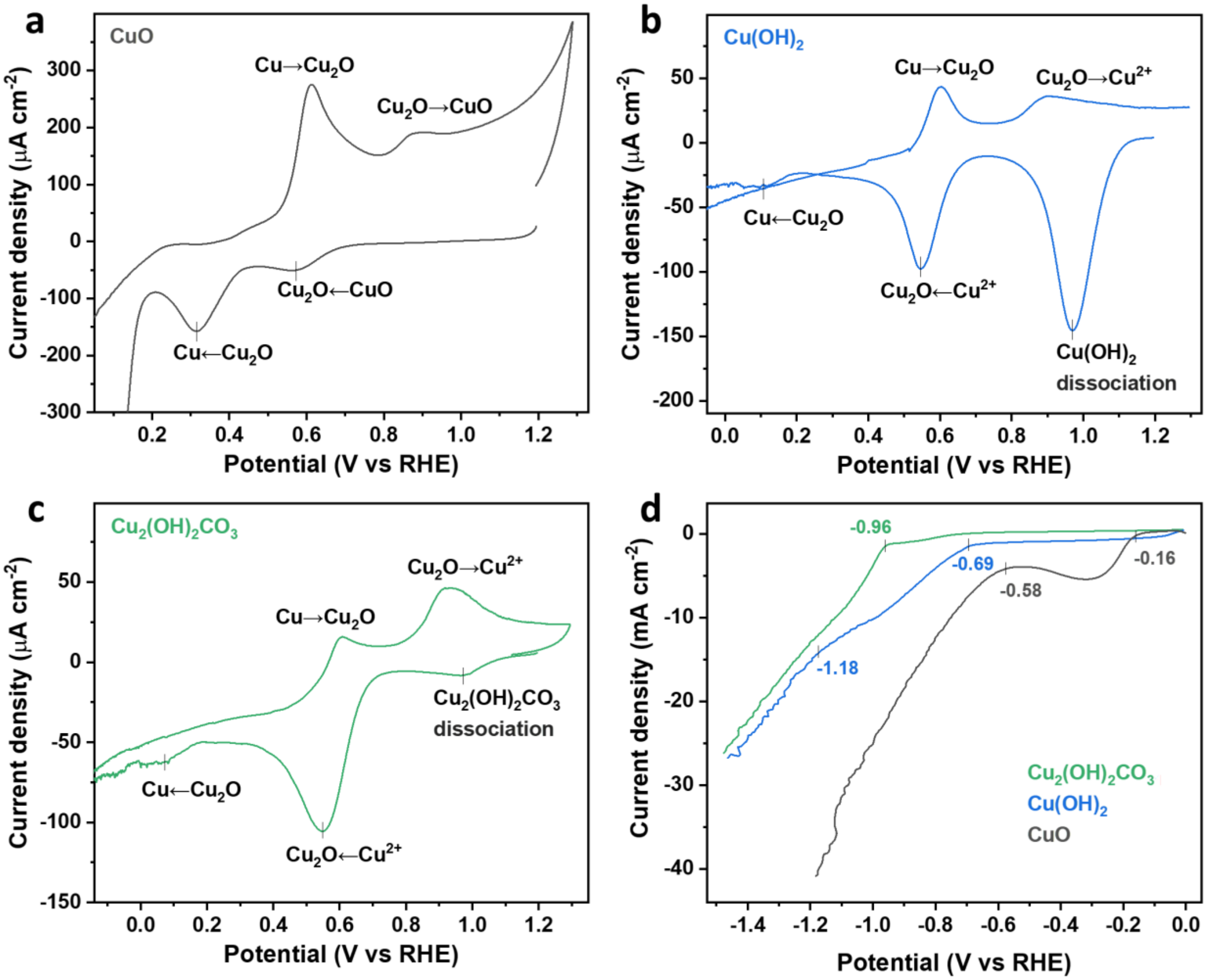

Supplementary Fig. 5. Cyclic Voltammetry (CV) of (a) CuO, (b) $Cu(OH)_2$, and (c) $Cu_2(OH)_2CO_3$ nanocrystals in 0.1 M KOH. (d) Linear Sweep Voltammetry (LSV) with these three catalysts in Ar-saturated 0.1 M $KHCO_3$. Potential sweep rate was 5 mV s^{-1}.

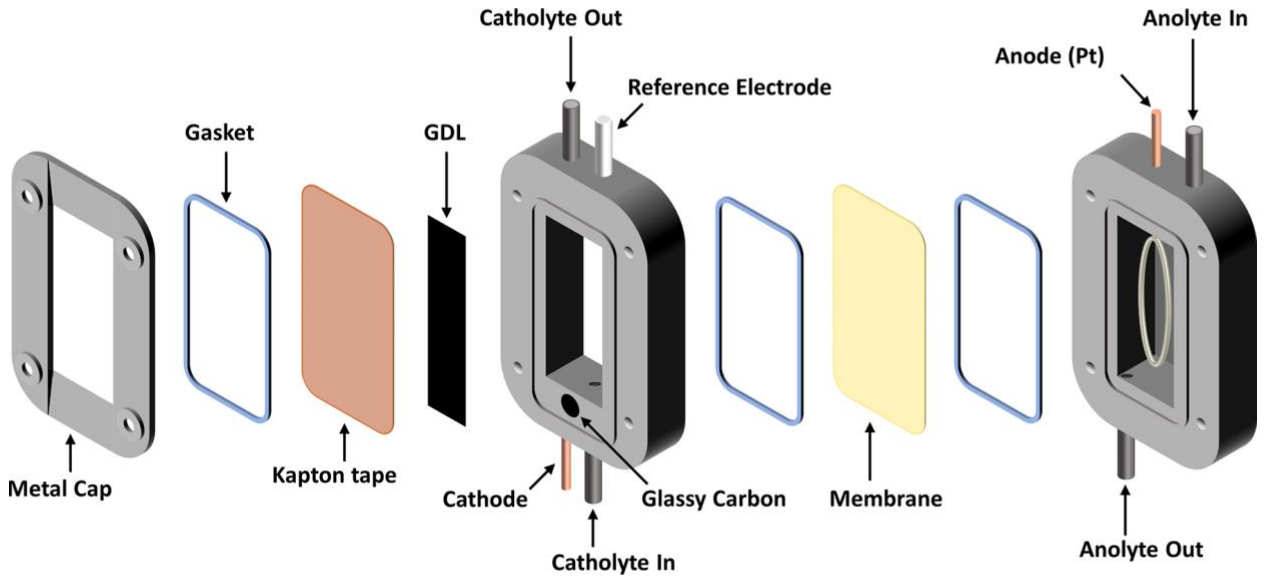

Supplementary Fig. 6. Exploded view of the customized cell for *operando* XRD characterization under CO_2RR conditions.

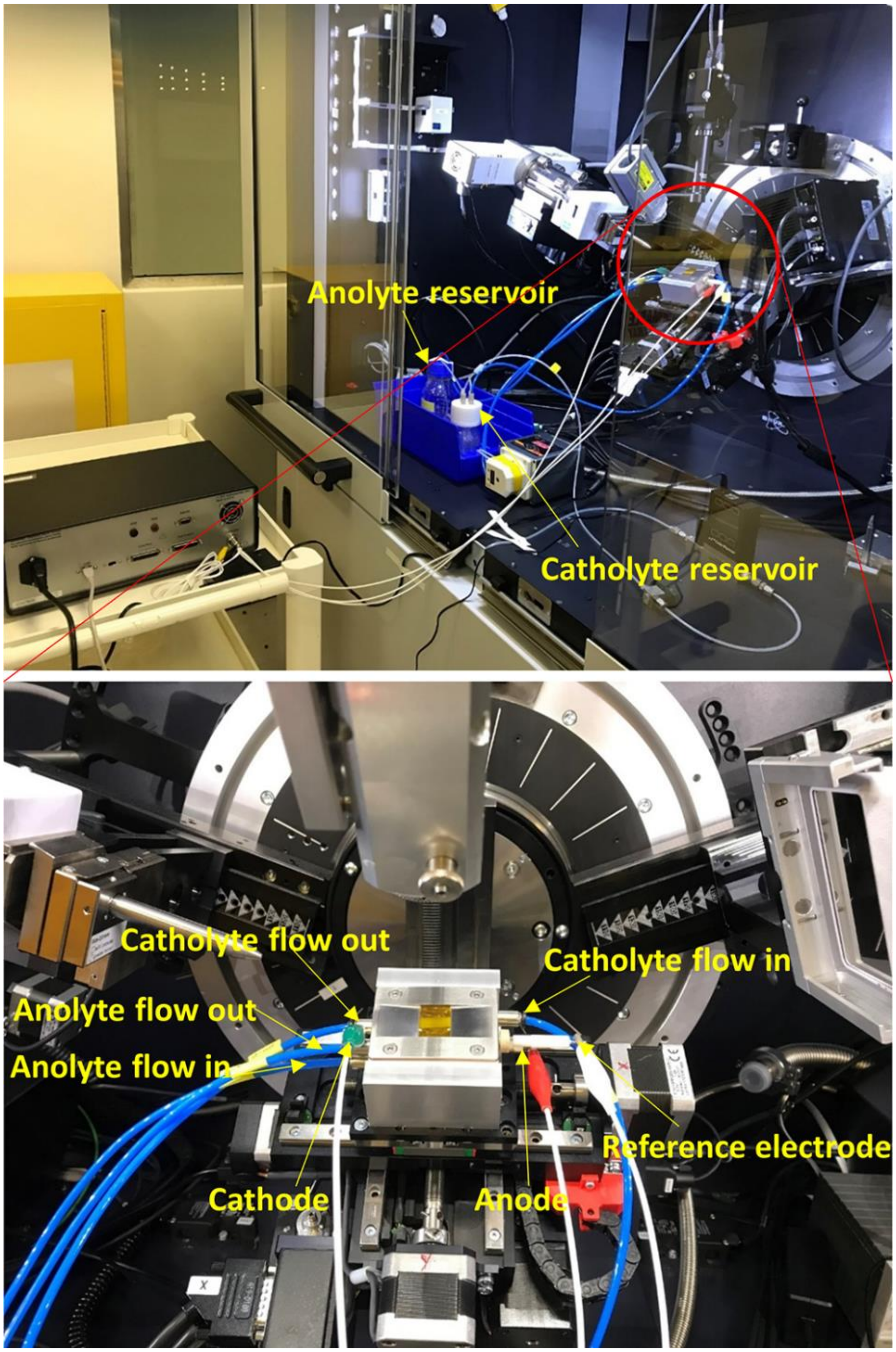

Supplementary Fig. 7. Setup for the *operando* XRD under the CO_2RR conditions.

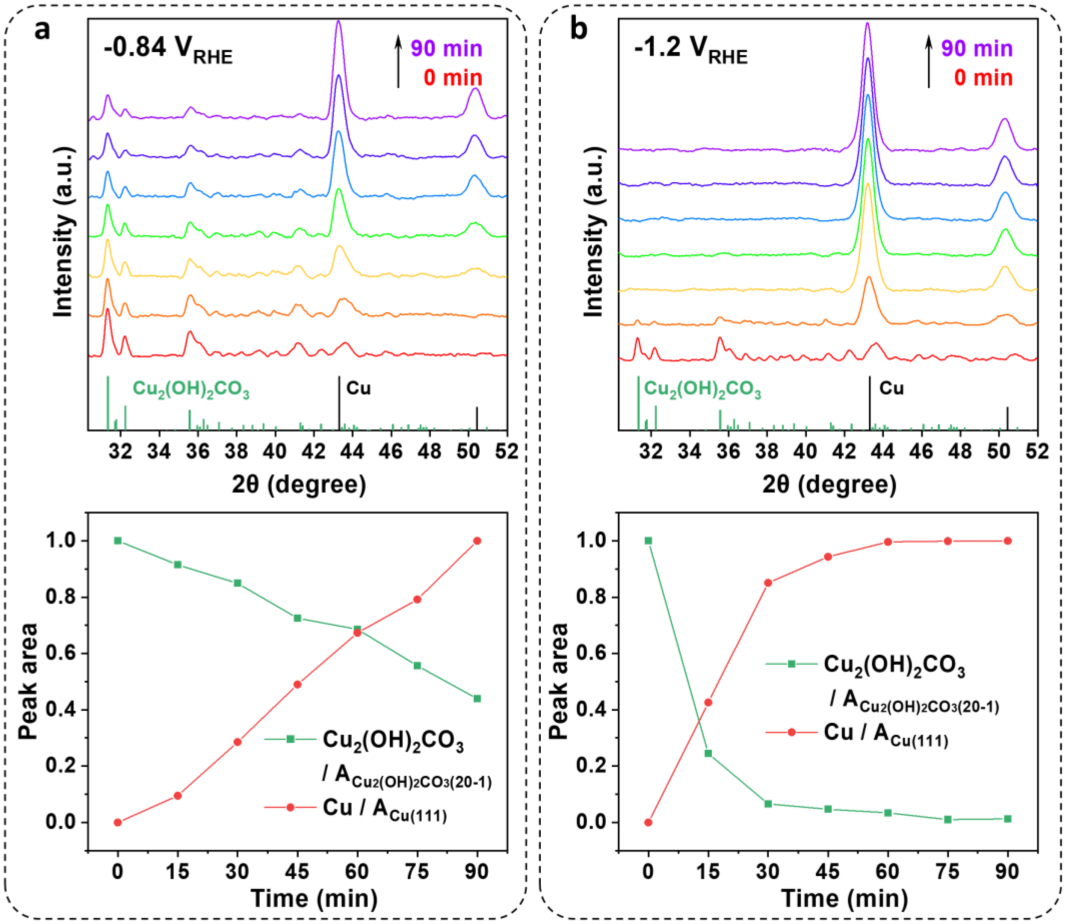

Supplementary Fig. 8. Time-resolved *operando* XRD patterns (upper panel) and the corresponding quantitative peak analysis (lower panel) for the CO_2RR with $Cu_2(OH)_2CO_3$ at (a) -0.84 V_{RHE} and (b) -1.2 V_{RHE}. Spectra were collected every 15 min. For the quantitative peak analysis, the integrated and normalized intensities of $Cu_2(OH)_2CO_3$(20-1) and Cu(111) were used.

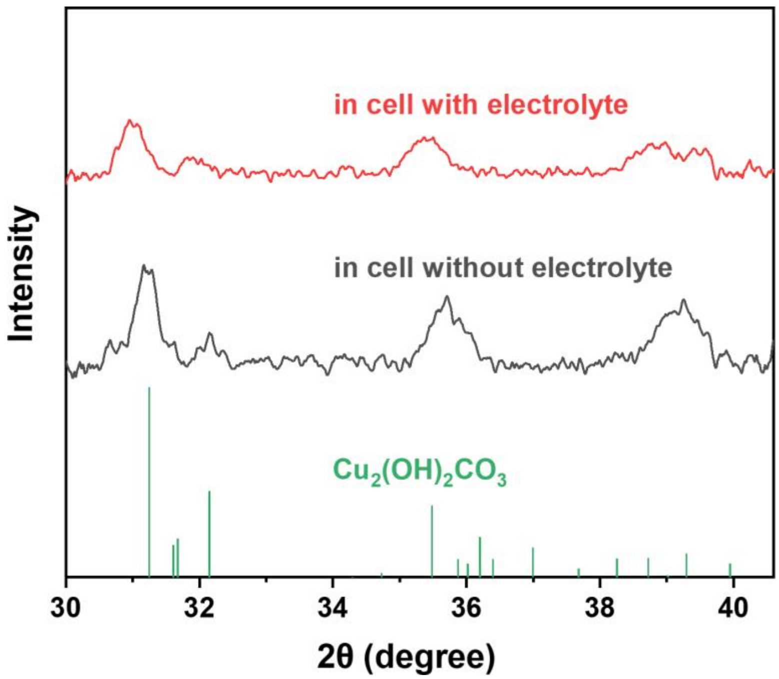

Supplementary Fig. 9. Comparison for the XRD patterns of $Cu_2(OH)_2CO_3$ electrode in fully assembled cell with and without electrolyte. Other parameters were kept same for the measurements.

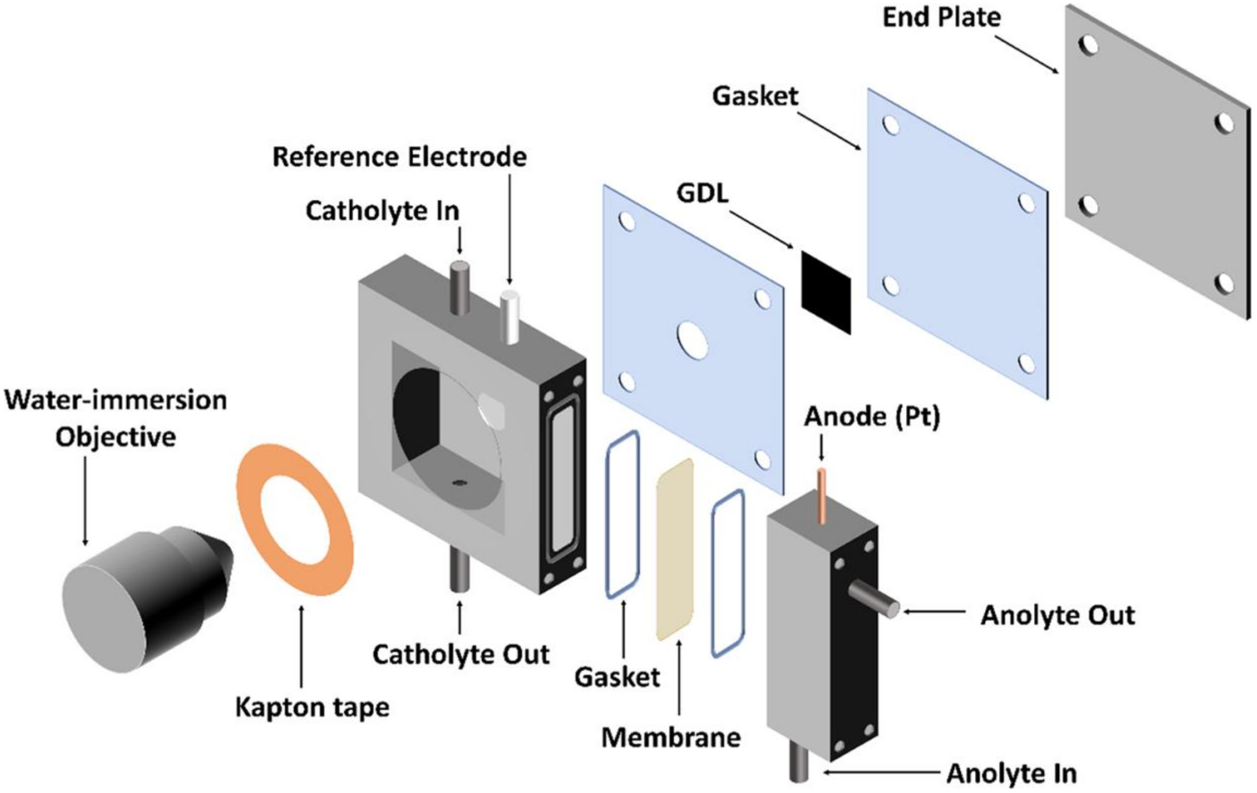

Supplementary Fig. 10. Exploded view of the customized cell for *operando* Raman characterization under CO_2RR conditions.

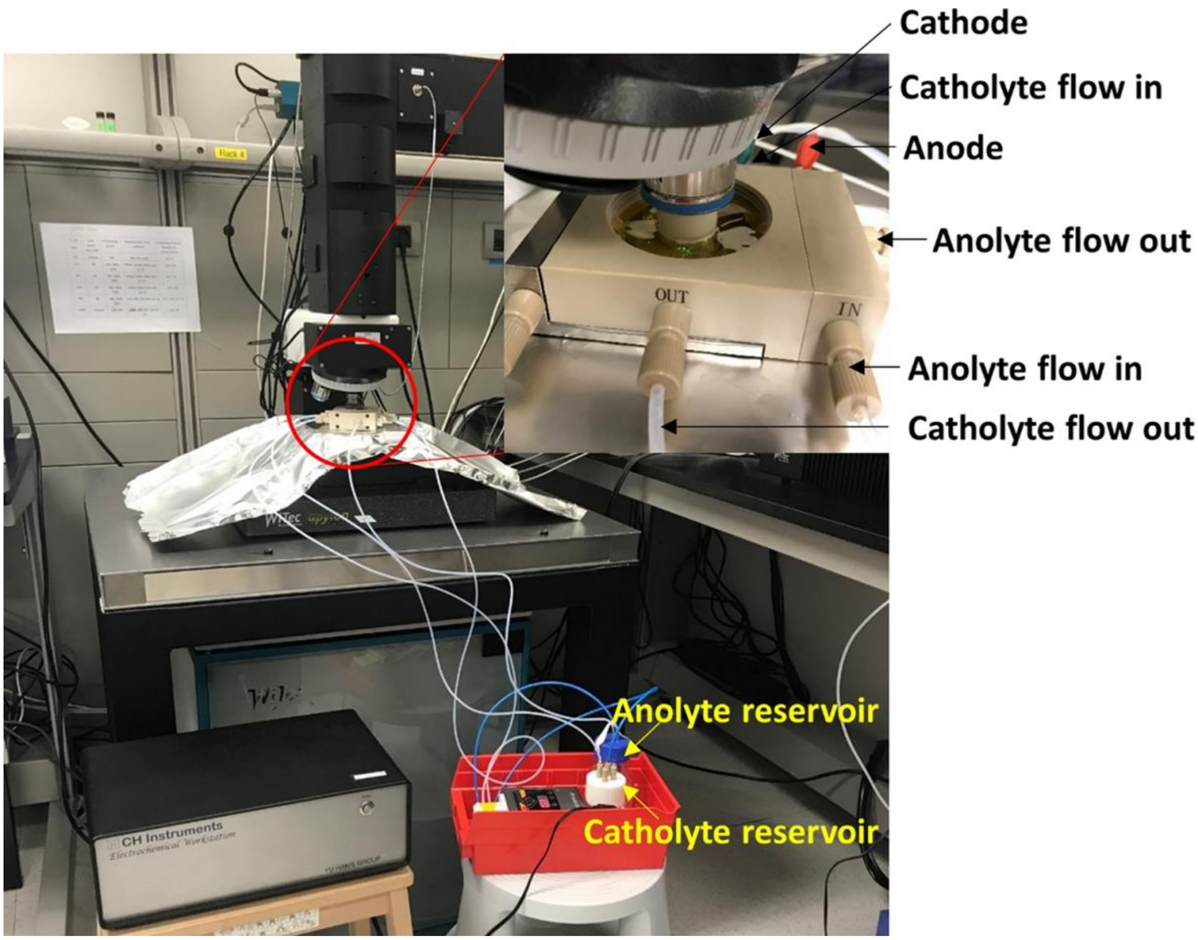

Supplementary Fig. 11. Setup for the *operando* Raman spectroscopy under the CO_2RR conditions.

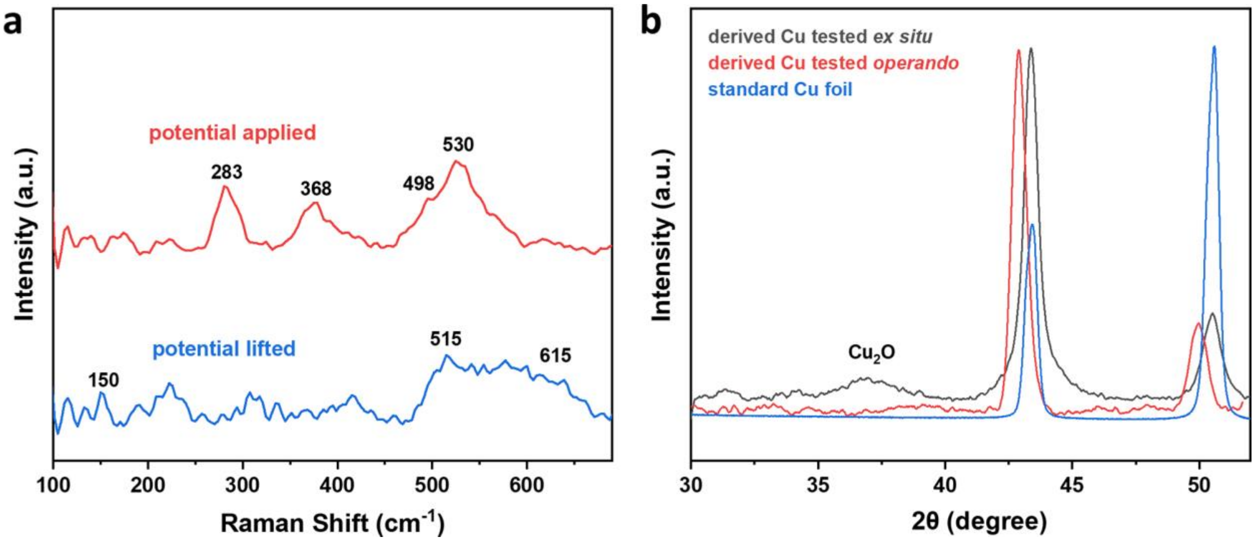

Supplementary Fig. 12. (a) Comparison for the Raman spectra collected when applying and then lifting the potential during the CO_2RR with CuO nanocrystals at -1.16 V_{RHE}. Other parameters were kept same for the measurements. (b) Comparison for the XRD patterns collected *operando* and *ex situ* (electrode removed from electrolyte and dried with N_2) during (or after) the CO_2RR with $Cu_2(OH)_2CO_3$ nanocrystals at -1.05 V_{RHE} for 1.5 h. Cu foil was tested on the same XRD instrument as a reference.

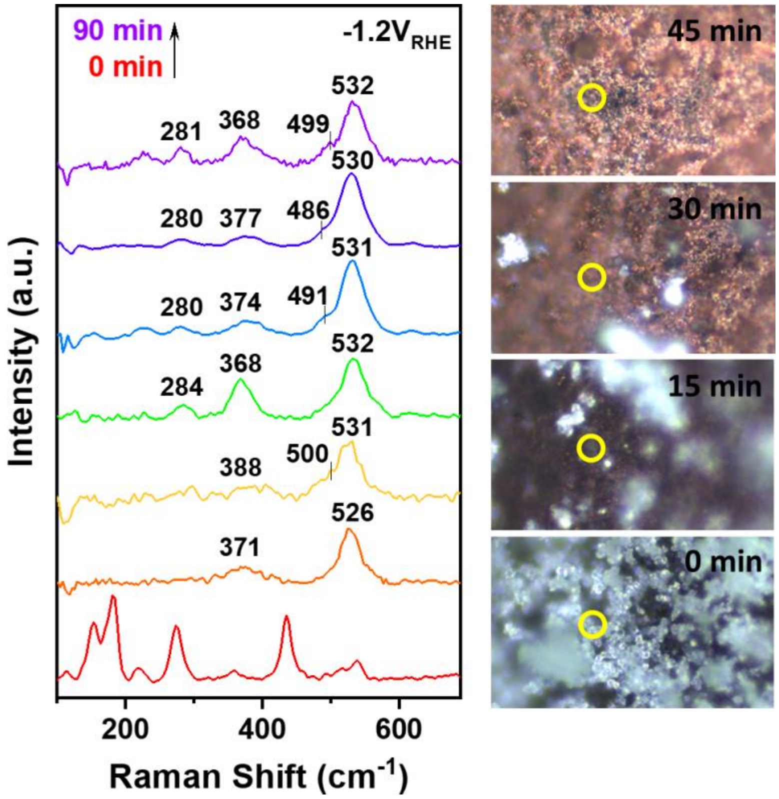

Supplementary Fig. 13. Time-resolved *operando* Raman spectra (left panel) and light microscopy (right panel) for the CO_2RR with $Cu_2(OH)_2CO_3$ at -1.2 V_{RHE}. Spectra were collected every 15 min.

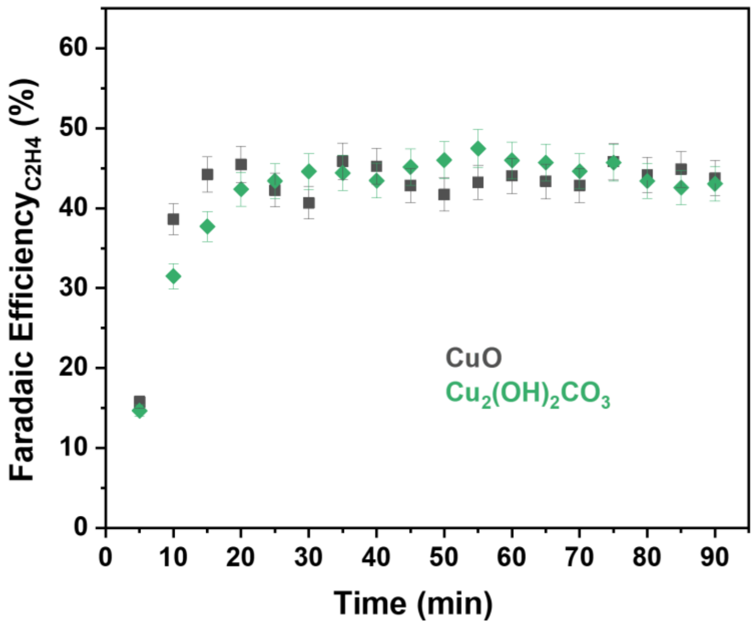

Supplementary Fig. 14. $FE_{C_2H_4}$ of the $Cu_2(OH)_2CO_3$ and CuO nanocrystals at -1.05 and -1.16 V_{RHE}, respectively. Both CO_2RR experiments were performed in an H-type cell using CO_2-saturated 0.1 M $KHCO_3$ as the electrolyte for 1.5 h. Error bars represent the standard deviation of three independent measurements.

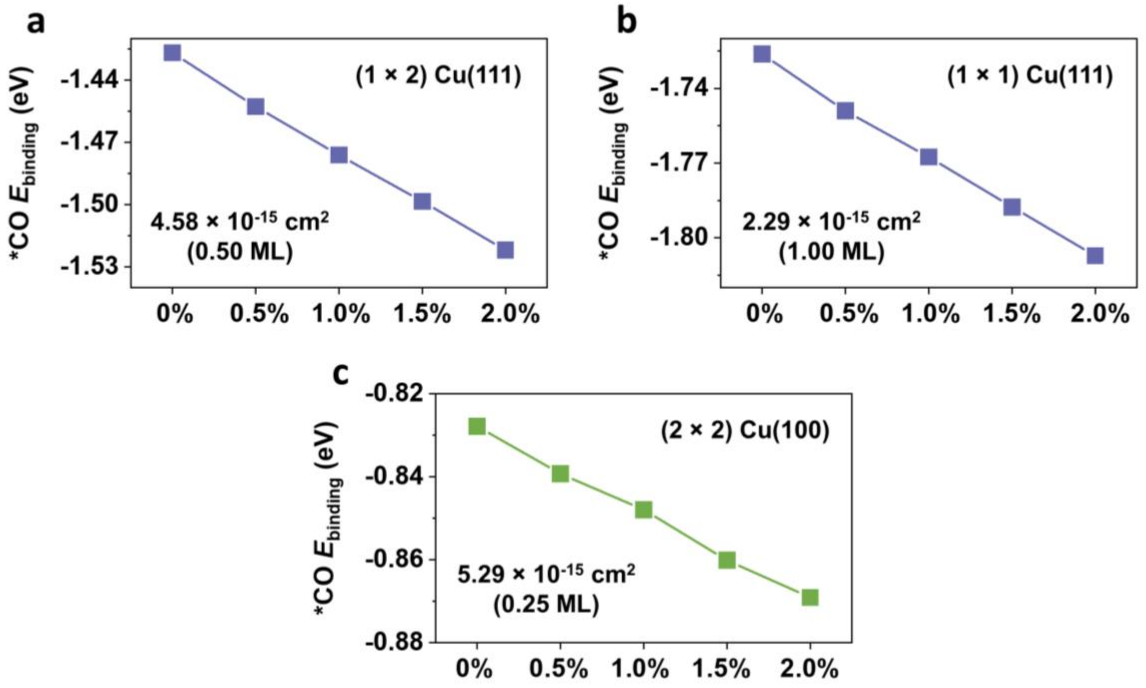

Supplementary Fig. 15. Calculated binding energies of *CO for (a) 1 × 2 Cu(111) unit cell, (b) 1 × 1 Cu(111) unit cell, and (c) 2 × 2 Cu(100) unit cell with different strain values (0.0%, 0.5%, 1.0%, 1.5% and 2.0%). The lateral dimension of the Cu unit cell is adjusted to increase the *CO coverage. For Cu(111), the *CO coverage is elevated from 0.25 ML with a cross-sectional area of 9.15×10^{-15} cm^2 (2 × 2 unit cell) to 0.50 ML (4.58×10^{-15} cm^2, 1 × 2 unit cell) and 1.00 ML (2.29×10^{-15} cm^2, 1 × 1 unit cell); for Cu(100), the *CO coverage is elevated from 0.11 ML with a cross-sectional area of 1.20×10^{-14} cm^2 (3 × 3 unit cell) to 0.25 ML (5.29×10^{-15} cm^2, 2 × 2 unit cell. These values are close to the reported cross-sectional area occupied by adsorbed CO (3.9×10^{-15} cm^2, assuming a roughness factor of unity).[4] For both Cu(111) and Cu(100), the strain-induced increase in *CO binding energy did not change significantly with the degree of the *CO coverage: 0.11, 0.10, and 0.08 eV for 2 × 2, 1 × 2, and 1 × 1 unit cells, respectively, for Cu(111); 0.03 and 0.04 eV for 3 × 3 and 2 × 2 unit cells, respectively, for Cu(100).

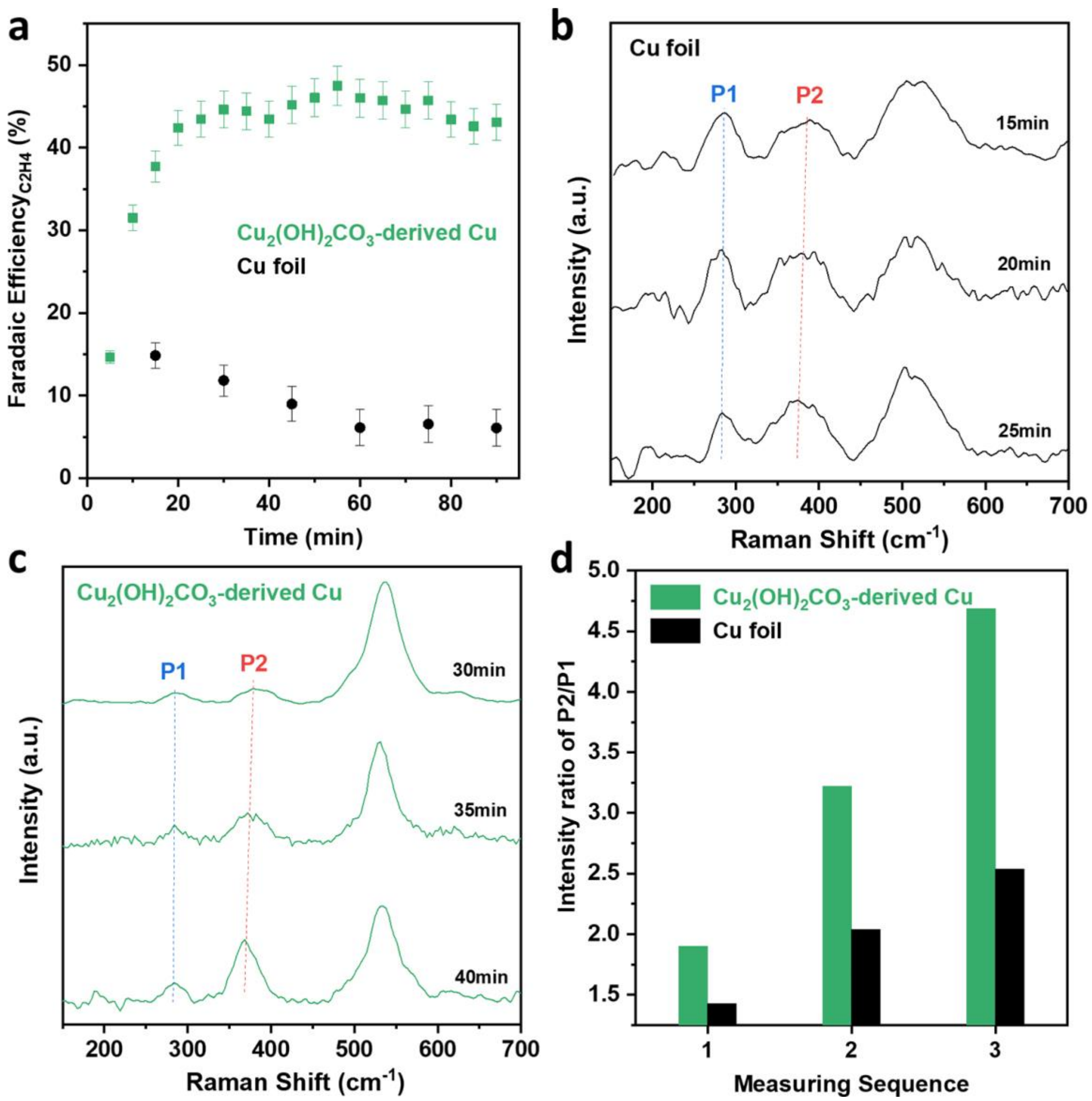

Supplementary Fig. 16. (a) $FE_{C_2H_4}$ of $Cu_2(OH)_2CO_3$-derived Cu at -1.05 V_{RHE} and Cu foil at -1.12 V_{RHE}. (b-c) *Operando* Raman spectra acquired on (b) Cu foil at -1.12 V_{RHE} and (c) $Cu_2(OH)_2CO_3$-derived Cu -1.05 V_{RHE} at different reaction times. (d) Intensity ratio of P2 to P1 summarized from (b) and (c). The CO_2RR experiments were performed using CO_2-saturated 0.1 M $KHCO_3$ as the electrolyte for 1.5 h. In the Raman spectra, the P1 and P2 bands correspond to the CO frustrated rotational mode and Cu–CO stretching vibrational mode, respectively, and the higher P2/P1 ratio can be used as an indicator of higher *CO coverage on the Cu surface.[5] Error bars represent the standard deviation of three independent measurements.

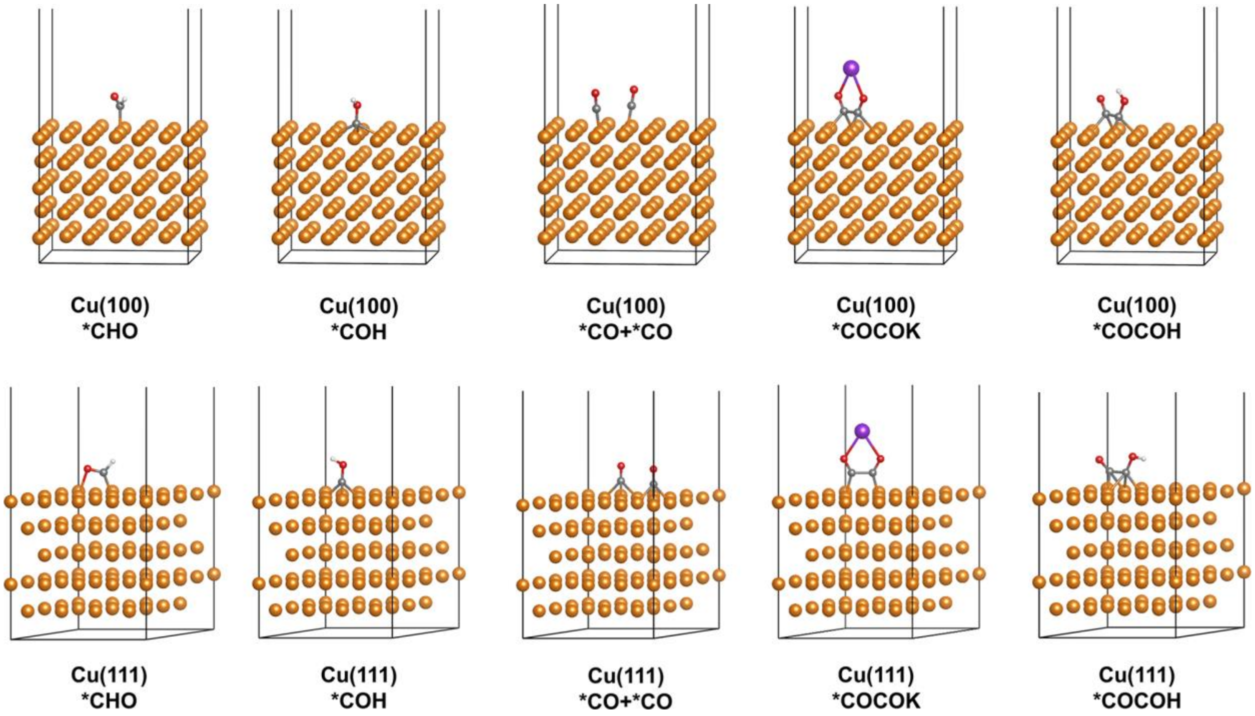

Supplementary Fig. 17. Optimized structural models of *CHO, *COH, *CO+*CO, *COCOK and *COCOH adsorbed on Cu(100) and Cu(111) surfaces before introducing lattice strain.

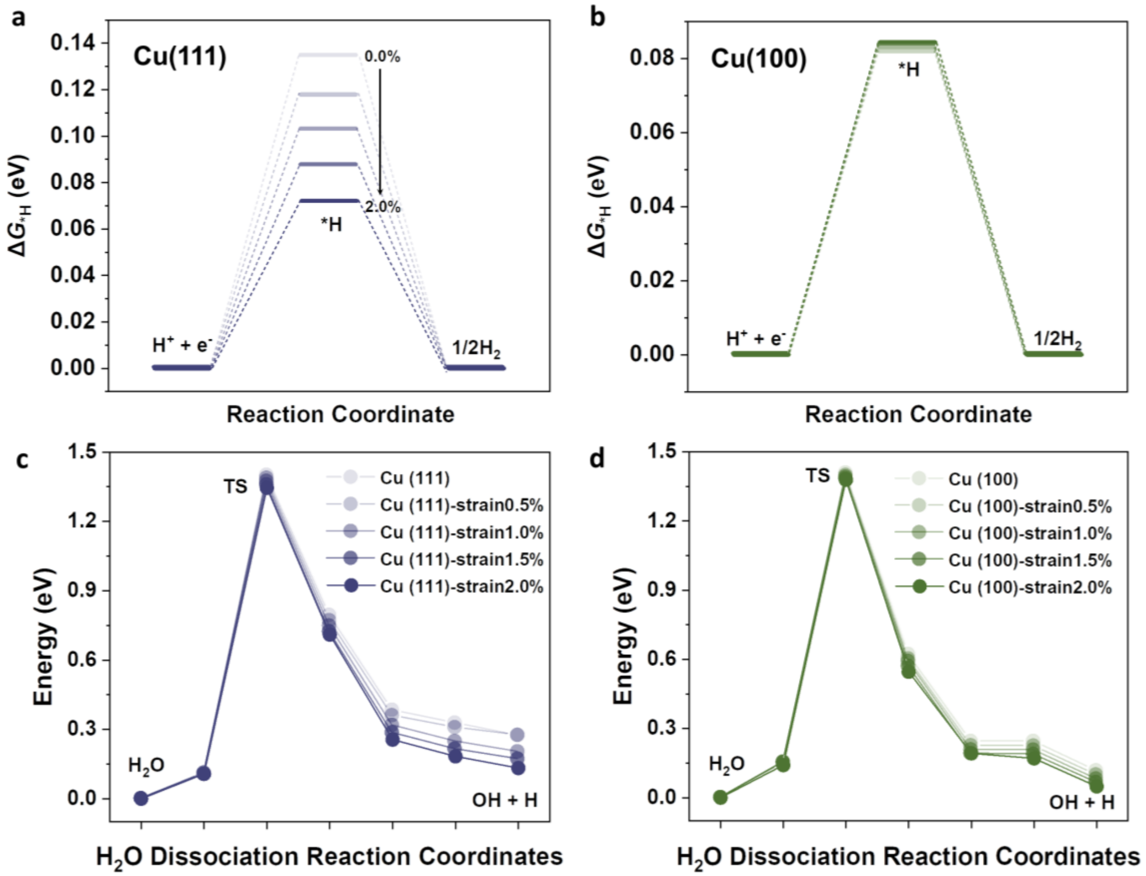

Supplementary Fig. 18. Calculated adsorption free energy of *H (ΔG_{*H}) and energy barriers of water dissociation on (a, c) Cu(111) and (b, d) Cu(100) with different strain values (0.0%, 0.5%, 1.0%, 1.5% and 2.0%).

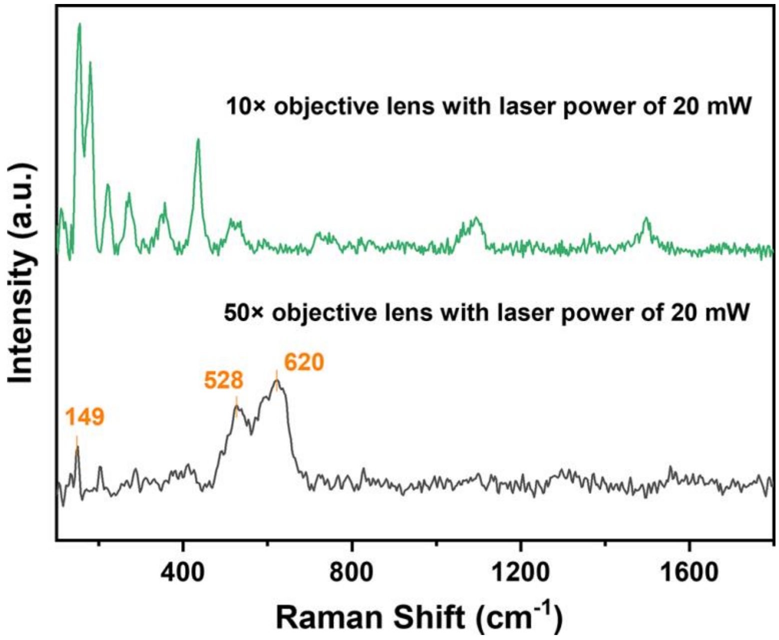

Supplementary Fig. 19. Raman spectra acquired with high (50x) and low (10x) magnification objective lenses during the *ex situ* Raman characterization for the $Cu_2(OH)_2CO_3$ nanocrystals. Other parameters were kept the same for the measurements.

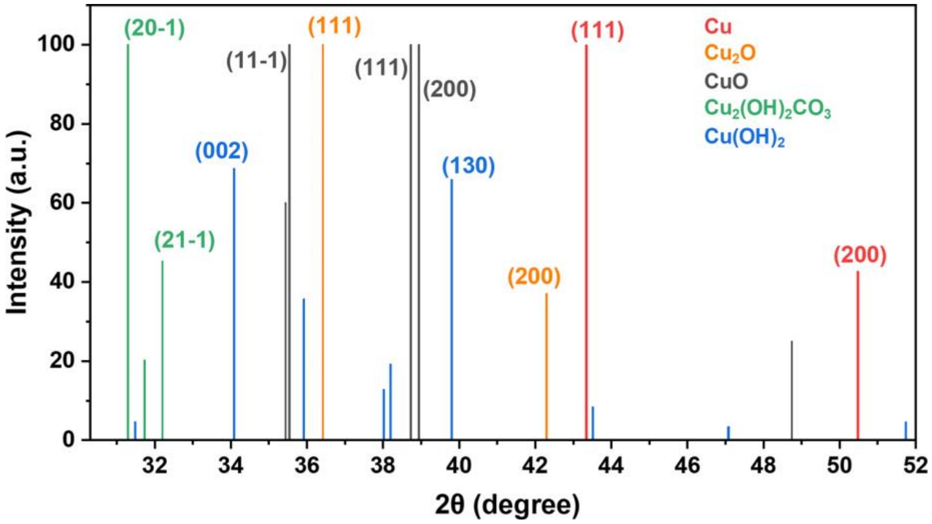

Supplementary Fig. 20. 2θ range of interest for the *operando* XRD measurements under CO_2RR conditions.

Supplementary Table 1. Determination of mean crystal size and electrochemically active surface area (ECSA) of the catalysts under study. The mean crystal sizes of nanocrystals were estimated using Scherrer equation, while that of Cu foil was estimated using the transmission electron microscopic image. Double layer capacitance (C_{dl}) was measured to determine the ECSA. The ECSA for electropolished Cu foil was defined as 1, and the geometric area of all electrodes were controlled to be the same.

**Sample**	**$Cu_2(OH)_2CO_3$**	**$Cu(OH)_2$**	**CuO**	**Cu foil**
Initial mean crystal size (nm)	24.7 ± 3.5	19 ± 0.5	10 ± 0.5	4500
Derived mean crystal size (nm)	11.8 ± 1.7	11.7 ± 1.4	11.6 ± 1.4	4500
C_{dl} of the (derived-) Cu electrode (mF cm^{-2})	2.53	2.10	2.85	0.16
ECSA of the (derived-) Cu electrode	16.16	13.70	17.27	1

Supplementary Table 2. Diffraction peak shifts and the corresponding lattice parameters and strain values observed during the *operando* XRD on $Cu_2(OH)_2CO_3$, $Cu(OH)_2$, and CuO nanocrystals at different CO_2RR potentials. Cu foil was tested on the same XRD instrument as a reference.

**Cu precursor**	**CO_2RR potential (V_{RHE})**	**Cu(111)**			**Cu(200)**		
		**Average XRD peak position (2θ)**	**Lattice parameter (Å)**	**Strain (%)**	**Average XRD peak position (2θ)**	**Lattice parameter (Å)**	**Strain (%)**
$Cu_2(OH)_2CO_3$	-0.84	43.1586	3.6305	**0.51**	50.1611	3.6373	**0.67**
	-1.05	43.1410	3.6319	**0.55**	50.1313	3.6394	**0.72**
	-1.20	43.1097	3.6345	**0.61**	50.1414	3.6387	**0.70**
$Cu(OH)_2$	-0.63	43.1953	3.6276	**0.42**	50.2687	3.6301	**0.46**
	-0.83	43.1751	3.6292	**0.47**	50.2565	3.6309	**0.49**
	-1.08	43.1912	3.6279	**0.43**	50.2526	3.6311	**0.49**
CuO	-0.82	43.3870	3.6123	**0.00**	50.5121	3.6137	**0.01**
	-1.16	43.3602	3.6145	**0.06**	50.4813	3.6158	**0.07**
	-1.23	43.3810	3.6128	**0.02**	50.4422	3.6184	**0.14**
Cu foil	-	43.3881	3.6123	**0**	50.5186	3.6133	**0**

Supplementary Table 3. Unit-cell volumes of the three oxidized Cu precursors, the corresponding cell volume reduction after their electroreduction to Cu, and the corresponding tensile strain of the derived Cu.

Sample	Unit-cell volume (Å^3)	Cu atoms per unit-cell	Cell volume per 4 Cu atoms (Å^3)	Cell volume reduction per 4 Cu atoms (%)	Strain (on (111) surface) (%)
$Cu_2(OH)_2CO_3$	364.35	8	182.175	74	0.55
$Cu(OH)_2$	164.14	4	164.14	71	0.43
CuO	81.08	4	81.08	42	0.06
Cu	47.16	4	47.16	-	-

Supplementary Table 4. Thermodynamic parameters for calculating Gibbs free energies of Cu(111) without strain (i.e., strain 0.0%).

**Cu(111) -strain 0.0%**	E_{DFT}	**ZPE**	$\int C_v dT$	$T\Delta S$	**G**
slab	-282.911				
*CO	-298.161	0.178	0.092	0.120	-298.012
*CHO	-301.037	0.444	0.132	0.159	-300.620
*COH	-300.697	0.452	0.115	0.117	-300.247
*CO+*CO	-313.783	0.343	0.238	0.306	-313.509
*COCO	-312.439	0.366	0.185	0.205	-312.093
*COCOH	-316.101	0.684	0.237	0.256	-315.437

Supplementary Table 5. Thermodynamic parameters for calculating Gibbs free energies of Cu(111)-strain 0.5%.

**Cu(111)-strain 0.5%**	E_{DFT}	**ZPE**	$\int C_v dT$	$T\Delta S$	**G**
slab	-282.568				
*CO	-297.845	0.179	0.092	0.122	-297.696
*CHO	-300.721	0.444	0.132	0.175	-300.320
*COH	-300.396	0.451	0.115	0.117	-299.947
*CO+*CO	-313.501	0.331	0.215	0.277	-313.232
*COCO	-312.127	0.370	0.210	0.268	-311.815
*COCOH	-315.799	0.661	0.213	0.262	-315.186

Supplementary Table 6. Thermodynamic parameters for calculating Gibbs free energies of Cu(111)-strain 1.0%.

**Cu(111)-strain 1.0%**	E_{DFT}	**ZPE**	$\int C_v dT$	$T\Delta S$	**G**
slab	-282.082				
*CO	-297.386	0.178	0.118	0.187	-297.277
*CHO	-300.264	0.442	0.132	0.175	-299.865
*COH	-299.951	0.448	0.117	0.119	-299.506
*CO+*CO	-313.073	0.346	0.237	0.297	-312.787
*COCO	-311.671	0.367	0.211	0.269	-311.363
*COCOH	-315.351	0.662	0.240	0.267	-314.716

Supplementary Table 7. Thermodynamic parameters for calculating Gibbs free energies of Cu(111)-strain 1.5%.

**Cu(111)-strain 1.5%**	E_{DFT}	**ZPE**	$\int C_v dT$	$T\Delta S$	**G**
slab	-281.447				
*CO	-296.777	0.171	0.092	0.121	-296.635
*CHO	-299.657	0.444	0.132	0.174	-299.255
*COH	-299.356	0.451	0.115	0.116	-298.906
*CO+*CO	-312.494	0.344	0.238	0.300	-312.212
*COCO	-311.067	0.367	0.185	0.205	-310.721
*COCOH	-314.754	0.665	0.239	0.308	-314.158

Supplementary Table 8. Thermodynamic parameters for calculating Gibbs free energies of Cu(111)-strain 2.0%.

**Cu(111)-strain 2.0%**	E_{DFT}	**ZPE**	$\int C_v dT$	$T\Delta S$	**G**
slab	-280.667				
*CO	-296.023	0.180	0.117	0.183	-295.908
*CHO	-298.905	0.445	0.132	0.173	-298.501
*COH	-298.616	0.451	0.116	0.117	-298.167
*CO+*CO	-311.770	0.347	0.237	0.292	-311.478
*COCO	-310.319	0.370	0.210	0.267	-310.006
*COCOH	-314.013	0.664	0.237	0.304	-313.415

Supplementary Table 9. Thermodynamic parameters for calculating Gibbs free energies of Cu(100) without strain (i.e., strain 0.0%).

**Cu(100)-strain 0.0%**	E_{DFT}	**ZPE**	$\int C_v dT$	$T\Delta S$	**G**
slab	-313.658				
*CO	-329.282	0.179	0.092	0.121	-329.133
*CHO	-332.066	0.427	0.108	0.152	-331.683
*COH	-332.180	0.451	0.143	0.152	-331.738
*CO+*CO	-344.850	0.361	0.208	0.299	-344.580
*COCO	-343.905	0.363	0.210	0.232	-343.564
*COCOH	-347.756	0.700	0.222	0.236	-347.070

Supplementary Table 10. Thermodynamic parameters for calculating Gibbs free energies of Cu(100)-strain 0.5%.

Cu(100)-strain 0.5%	E_{DFT}	ZPE	$\int C_v dT$	$T\Delta S$	G
slab	-313.232				
*CO	-328.863	0.173	0.093	0.128	-328.725
*CHO	-331.729	0.440	0.106	0.122	-331.305
*COH	-331.773	0.445	0.117	0.112	-331.324
*CO+*CO	-344.449	0.357	0.210	0.308	-344.190
*COCO	-343.501	0.353	0.183	0.188	-343.153
*COCOH	-347.353	0.700	0.222	0.236	-346.667

Supplementary Table 11. Thermodynamic parameters for calculating Gibbs free energies of Cu(100)-strain 1.0%.

Cu(100)-strain 1.0%	E_{DFT}	ZPE	$\int C_v dT$	$T\Delta S$	G
slab	-312.634				
*CO	-328.273	0.179	0.092	0.121	-328.123
*CHO	-331.146	0.446	0.131	0.170	-330.739
*COH	-331.187	0.442	0.118	0.114	-330.741
*CO+*CO	-343.869	0.359	0.210	0.306	-343.607
*COCO	-342.928	0.354	0.186	0.186	-342.574
*COCOH	-346.778	0.700	0.222	0.235	-346.091

Supplementary Table 12. Thermodynamic parameters for calculating Gibbs free energies of Cu(100)-strain 1.5%.

**Cu(100)-strain 1.5%**	E_{DFT}	**ZPE**	$\int C_v dT$	$T\Delta S$	**G**
slab	-311.881				
*CO	-327.526	0.180	0.117	0.183	-327.412
*CHO	-330.405	0.447	0.131	0.169	-329.997
*COH	-330.444	0.443	0.118	0.113	-329.997
*CO+*CO	-343.133	0.361	0.235	0.369	-342.906
*COCO	-342.196	0.352	0.187	0.189	-341.846
*COCOH	-346.047	0.700	0.222	0.244	-345.370

Supplementary Table 13. Thermodynamic parameters for calculating Gibbs free energies of Cu(100)-strain 2.0%.

**Cu(100)-strain 2.0%**	E_{DFT}	**ZPE**	$\int C_v dT$	$T\Delta S$	**G**
slab	-310.976				
*CO	-326.628	0.180	0.092	0.121	-326.477
*CHO	-329.512	0.439	0.107	0.121	-329.087
*COH	-329.549	0.442	0.118	0.114	-329.103
*CO+*CO	-342.243	0.355	0.184	0.243	-341.948
*COCO	-341.314	0.363	0.183	0.228	-340.996
*COCOH	-345.165	0.699	0.222	0.236	-344.479

References

(1) Böhme, A.; Sterl, F.; Kath, E.; Ubl, M.; Manninen, V.; Giessen, H. Electrochemistry on Inverse Copper Nanoantennas: Active Plasmonic Devices with Extraordinarily Large Resonance Shift. *ACS Photonics* **2019,** *6*, 1863-1868.

(2) Caballero-Briones, F.; Artés, J. M.; Díez-Pérez, I.; Gorostiza, P.; Sanz, F. Direct Observation of the Valence Band Edge by in Situ ECSTM-ECTS in p-Type Cu_2O Layers Prepared by Copper Anodization. *Journal of Physical Chemistry C* **2009,** *113*, 1028-1036.

(3) Henckel, D. A.; Counihan, M. J.; Holmes, H. E.; Chen, X. Y.; Nwabara, U. O.; Verma, S.; Rodríguez-López, J.; Kenis, P. J. A.; Gewirth, A. A. Potential Dependence of the Local pH in a CO_2 Reduction Electrolyzer. *ACS Catalysis* **2021,** *11*, 255-263.

(4) Hori, Y.; Murata, A.; Yoshinami, Y. Adsorption of CO, intermediately formed in electrochemical reduction of CO_2, at a copper electrode. *Journal of the Chemical Society, Faraday Transactions* **1991**, *87*, 125-128.

(5) Zhan, C.; Dattila, F.; Rettenmaier, C.; Bergmann, A.; Kühl, S.; Garcia-Muelas, R.; López, N.; Cuenya, B. R. Revealing the CO Coverage-Driven C-C Coupling Mechanism for Electrochemical CO_2 Reduction on Cu_2O Nanocubes via Operando Raman Spectroscopy. *ACS Catalysis* **2021,** *11*, 7694-7701.
